# Learning Actions From Natural Language Instructions Using an ON-World Embodied Cognitive Architecture

**DOI:** 10.3389/fnbot.2021.626380

**Published:** 2021-05-13

**Authors:** Ioanna Giorgi, Angelo Cangelosi, Giovanni L. Masala

**Affiliations:** ^1^Department of Computer Science, The University of Manchester, Manchester, United Kingdom; ^2^Department of Computing and Mathematics, Manchester Metropolitan University, Manchester, United Kingdom

**Keywords:** cognitive architecture, natural language learning, language to action, semantic mapping, abstract words, action grounding, robot action, developmental cognitive robotics

## Abstract

Endowing robots with the ability to view the world the way humans do, to understand natural language and to learn novel semantic meanings when they are deployed in the physical world, is a compelling problem. Another significant aspect is linking language to action, in particular, utterances involving abstract words, in artificial agents. In this work, we propose a novel methodology, using a brain-inspired architecture, to model an appropriate mapping of language with the percept and internal motor representation in humanoid robots. This research presents the first robotic instantiation of a complex architecture based on the Baddeley's Working Memory (WM) model. Our proposed method grants a scalable knowledge representation of verbal and non-verbal signals in the cognitive architecture, which supports incremental open-ended learning. Human spoken utterances about the workspace and the task are combined with the internal knowledge map of the robot to achieve task accomplishment goals. We train the robot to understand instructions involving higher-order (abstract) linguistic concepts of developmental complexity, which cannot be directly hooked in the physical world and are not pre-defined in the robot's static self-representation. Our proposed interactive learning method grants flexible run-time acquisition of novel linguistic forms and real-world information, without training the cognitive model anew. Hence, the robot can adapt to new workspaces that include novel objects and task outcomes. We assess the potential of the proposed methodology in verification experiments with a humanoid robot. The obtained results suggest robust capabilities of the model to link language bi-directionally with the physical environment and solve a variety of manipulation tasks, starting with limited knowledge and gradually learning from the run-time interaction with the tutor, past the pre-trained stage.

## Introduction

To enable natural human-robot cooperation, robots must allow humans to tell them what to do, guide them how to do it and shape their learning and understanding of concepts on their surroundings, using natural language. Efficient human-robot interaction depends on how well-robots can understand natural language, how they translate natural language commands into sensorimotor exploration and, according to Cantrell et al. (2011), whether they can acquire and semantically ground new natural language constructions at run-time. Knowledge pre-programming in robotic architectures requires a significant vocabulary of concepts and bodily functions to be pre-trained for robots to be tasked in natural ways. Moreover, it cannot account for novel encounters not anticipated at design time that emerge from real-world exchanges with humans (Cantrell et al., 2011).

Cangelosi and Schlesinger (2018) claim that the physical body is instrumental for the development of human-like intelligence in robots, starting from child psychology. They reason that the embodied agent should regulate the interactions among body, brain, and environment, to acquire bodily and mental capabilities in its physical surroundings. Therefore, we motivate that non-embodied natural language processing (NLP) solutions are not suitable in this context. Moreover, NLP systems do not address language acquisition, which is important for the understanding of the natural language instructions, they require a large amount of training data and, are unable to explain their outputs, in particular those related to the external environment and the body. Intuitively, effective embodied system should be able to map verbal language autonomously to the perceived workspace of concrete objects and actions and, understand more abstract expressions of language that cannot be directly mapped to the physical world. Moreover, they should adapt to the dynamics of the environment to learn new concepts or behaviours at run-time, i.e., out of the pre-trained state and, apply this knowledge directly to solve tasks.

Kurup and Lebiere (2012) argue that human cognitive modelling in robots for general problem solving is best achieved via the use of cognitive architectures. These architectures intend to match the current state of the world to the content, recall relevant knowledge from previous solutions stored away in some form and apply that knowledge to the current task by modifying previous solutions accordingly. Cognitive capabilities needed for human intelligence, like perception, language, and decision-making are currently studied independently as a function or crafted algorithms. However, relevant studies clearly state the link between biological findings and language processing (Palm, 1990; Pulvermüller and Garagnani, 2014). Golosio et al. (2015) also argue that such cognitive functionalities can be validated through a bio-inspired architecture using artificial neural networks. Seminal works (Golosio et al., 2015; Giorgi et al., 2020) have proven its capability on verbal language acquisition and development, serving as a robust solution for language-related tasks. Giorgi et al. (2020) have demonstrated the ability of the cognitive architecture to parse and acquire multiple natural languages, with successful disambiguation in semantic and grammatical level.

In this work, we use this architecture (Golosio et al., 2015) as a framework to draw a novel methodology that addresses our specific research aim. We justify the use of this framework, given its compliance with a well-understood theoretical model of the human memory for language development in cognitive psychology (Cowan, 1998; Baddeley, 2012). *The major aim of this work is to address the acquisition of the meaning of primitive and higher-order semantic forms and how these meanings can be appropriately mapped to the physical environment of the robot*. Although several approaches have been proposed in this context (section Related Works), they have mainly developed learning models trained and able to generalise on a (considerable) pre-defined corpus, with limited run-time scalability on novel vocabulary not comprised in that corpus. *Therefore, our crux research aim, that is the acquisition of the meaning of words, is formulated and tackled in an incremental open-ended manner*. More specifically, we attempt to model a cognitive agent that is not dependant on the pre-trained taxonomic knowledge for anticipated scenarios, but rather can continue to acquire (amend and expand) such knowledge when it is brought to interaction in unknown real-world workspaces and, use the knowledge appropriately to attain a certain (novel) goal or outcome. With the help of human guidance, the agent first observes the new workspace to learn the objects contained in the workspace and what actions can be executed upon them, by self-mapping their percept or motor representations (perceived meaning) to the verbal utterances spoken by the human (semantic forms). The acquired primitive skills are then retrieved to construct external high-order commands that are not pre-defined in the static internal representation of the robot. Human involvement is important for this cognitively plausible setting. The tutor guides and loosely supervises the interactive, incremental learning of the system through verbal descriptions of the workspace (objects/tools) and step-by-step guided operations (actions), rather than representing high-level actions as the desired goal state (She et al., 2014), in a way that is similar to child-training and understandable by non-programmer humans.

We implemented novel procedural learning mechanisms, new knowledge representation of symbolic and sub-symbolic signals and, memory retrieval processes, in the existing framework, to acquire and elaborate this multimodal information in an uninterrupted open-ended manner. *Our novel contribution, which differs from other research that exploits this architecture (Golosio et al., 2015, Giorgi et al., 2020), can be summarised as follows:*

1. The framework was initially developed to study the procedural mechanisms involved in the elaboration and reproduction of verbal language only (Golosio et al., 2015). Differently, we do not model language learning as a primary goal, but rather how linguistic concepts emerge when natural language is hooked in the robot's physical world for task-solving being the desired outcome. Vocabulary, grammar, and syntactic and semantic soundness are yielded as a natural consequence of the human-guided interaction between language and action. This research presents the first robotic instantiation of a complex language architecture based on the Baddeley's Working Memory (WM) model (Baddeley, 2012). We extend the procedural learning mechanisms of the model, to grant functionalities of information elaboration that integrates language with representation forms of perception and action. The goal is to learn how to map semantic forms to the perceived meanings from vision and motors, starting from primitive concepts (direct link) to more abstract concepts. The latter can be indirectly mapped to the physical world by transferring the links for primitive concepts—internal state of the robot—through verbal explanations on how these links are combined to construct an abstract concept—human tutor.
2. To achieve our aim on the acquisition of semantic meanings from the environment, continuously and on a scale-up corpus not anticipated in the model pre-training, we implemented new mechanisms of knowledge representation in and retrieval from the long-term memory. This enables the architecture to learn continuously, to gradually expand its internal knowledge with information and meanings from novel workspaces and to apply this newly-acquired knowledge directly on-demand.
  Language constructions, here referring to the linguistic definition as the group of words forming a phrase (grammar, syntax), are therefore acquired as the robot operates in its environment and converts natural language commands into action and/or partial language production. Finally, we implemented a learning feedback in the model, which enables the robotic system to infer the lack of or inconsistency of information during task-solving, to initiate a request for auxiliary input from the interlocutor and, to actively reuse the provided information to continue working on the task.

The remainder of the paper is organised as follows. In section Related Works, we review the body of literature on grounding language into perception and action. In section Materials and Methods, we describe the bio-inspired framework used to draw our method and our final robotic system. The contributed methodology is extensively explained in section The Proposed Learning Methodology. The robot learning experiments are described in section The Robot Learning Experiments. Section Handling Sensor-based Uncertainty discusses sensory-based error handling. The obtained results are presented in section Results and outcomes are further discussed in section Discussion. Section Conclusions summarises the paper.

## Related Works

Findings in cognitive and developmental psychology, cognitive linguistics, and neuroscience emphasise the role of the embodied and grounded approach to the modelling of cognition in artificial agents (Wilson, 2002; Pecher and Zwaan, 2005). Assorted computational modelling methodologies of language grounding in developmental robotics (Cangelosi, 2010) and models of integration of sensorimotor and cognitive capabilities (Steels, 2003; Feldman and Narayanan, 2004; Perlovsky, 2009) strongly comply with this empirical and theoretical evidence. Our study also adheres to the embodied approach of integrating language and behaviour, using supervised learning.

Seminal contributions have been made to link language to perception and robot behaviour, by training the appropriate acquisition of language-meaning relationships from experience and, the ability to generalise the acquired relations in novel real-world encounters. We address these approaches below.

### Language Grounding in Visual Perception

The grounding of language into perception, action, and mental simulations has been extensively addressed in several works (Glenberg and Kaschak, 2002; Borghi et al., 2011). There have been seminal advances on grounding language to visual perception (Matuszek et al., 2012; Yu and Siskind, 2013; Tellex et al., 2014; Yang et al., 2016). A recent natural language processing system has successfully proven to describe in words the content of pictures (Arandjelovic and Zisserman, 2017). However, in their work, the acquisition of language and language grounding are not addressed and huge pairs of images are required to train this skill. Sabinasz et al. (2020) proposed a neural dynamic architecture based on recurrent neural networks to ground spatial language in perception, through a sequential combination of concepts. Their model can evolve in continuous time, which helps solve the grounding task without algorithmic control. Another research demonstrates a noise-resistant algorithm based on a hierarchical cognitive architecture implemented in a real-world robotic scenario with the iCub robot (Štepánová et al., 2018). They train visual-to-language mappings in an unsupervised manner using a fixed grammar.

### Language Grounding in Sensorimotor Representation

There is a body of research on linking natural language to sensorimotor representations. We focus on studies that comply with the notion of embodied cognition. Numerous works address language acquisition and grounding for the purpose of engineering applications. Probabilistic models are used to emerge symbols from raw data, like speech (Iwahashi, 2008), video and motion, to acquire visually grounded vocabulary (Roy and Pentland, 2002), the meaning of manipulation verbs (Roy, 2003) and their grounding in motor commands for conversational service robots (Roy et al., 2003) and, online learning of concepts and words (Araki et al., 2012). Other studies train real robots for manipulation learning, using reinforcement learning techniques (Levine et al., 2016; Gu et al., 2017). However, reinforcement learning highly focuses on designing specific functionalities in robots and does not grant a robust solution for general problem-solving. Moreover, the learning process is not fully understood and does not account the language, which is an instrumental aspect of human-robot cooperation. Action learning in robots has also been attempted by combining verbal descriptions from the web with visual features extracted from images, to explore tasks like setting a table (Dubba et al., 2014) and making a pancake (Beetz et al., 2011). These works adopt an unsupervised learning approach.

A contrasting viewpoint is the supervised learning approach, in which linguistic data and expected behaviour are predetermined by humans and used as ground truth to train their models to achieve self-organisation of language-meaning representations. Many studies have investigated the modelling of human language in robotic architectures (Sugita and Tani, 2005; Ogata et al., 2007; Yamada et al., 2015, 2016; Tani, 2016; Heinrich and Wermter, 2018; Hinaut and Twiefel, 2019; Recupero and Spiga, 2019). They use neural networks to model the integration of language with robot behaviour and validate the generalisation skills in robot learning experiments. Alike our work, they investigate language learning and representation in the robot's physical workspace. Moreover, Sugita and Tani (2005), Hinaut and Wermter (2014), Hinaut and Twiefel (2019), Moulin-Frier et al. (2017), and Mealier et al. (2017) also adopt a cognitive linguistic perspective to model usage-based language acquisition and production. Many of these works have successfully achieved humanlike intelligence in real robots (Ogata et al., 2005; Yamada et al., 2015, 2016; Tani, 2016) using recurrent neural networks. Yamada et al. (2016) investigate and implement a complex representation learning of language-motor mappings. Most of these models perform unidirectionally. Our approach aims to map language commands to actions and perceived actions to language, as in the embodied neurocognitive model of Heinrich et al. (2020). Other contribution has been made to address language learning (Morse and Cangelosi, 2017), motor learning (Demiris and Khadhouri, 2006), and affordance learning (Stoytchev, 2008; Jamone et al., 2018), by successful integration of language and the physical body. On the pitfall, these works mostly focus on individual functions and the architectures for learning have been designed as *ad-hoc* solutions for specific tasks.

In our current study, we therefore aim to (a) model the acquisition of concrete and abstract concept meanings, (b) using a complex language model over *ad-hoc* solutions and at-random exploration of language. (a) Similarly, Stramandinoli et al. (2012) have implemented a neuro-robotic model, which develops linguistic skills of abstract concepts, by first learning to map basic words to their motor exploration and then link higher-order actions to words, by connecting them indirectly with the basic words. They assess whether the neural controller can select and activate the appropriate sequence of motor actions for a given abstract word. Their method allows to freely add novel words or re-arrange the current associations; however, this requires re-training the neural model to include non-anticipated words. In our work, such concepts are not pre-trained, but acquired naturally by learning directly from the human after the robot is deployed in the real world. Moreover, the same abstract concepts can have multiple associations. An interesting work on abstract concepts studies the grounding of logic words “not,” “and,” and “or” (Yamada et al., 2017), which is little explored in current conventional works. Here, we do not account this kind of abstraction. (b) A complex unified framework, which integrates multiple modules, for concept and language learning, knowledge acquisition and decision-making has been proposed by Miyazawa et al. (2019). However, their model suffers from two main limitations. The use of parametric Bayesian modules requires the number of classes to be defined in advance, which does not support open-ended learning by the robot. Moreover, the architecture cannot be used to verify human cognitive functions. According to Baddeley (2012) and Cowan et al. (2012), such cognitive functions are better modelled using a working memory framework, from which the cognitive model used here takes its inspiration, as we will argue in the next sessions. Our contributed system in this study is the first robotic instantiation of a complex neural model based on Baddeley's architecture.

### Interactive Learning in Changing Environments

One important aspect, which generally lacks in the body of literature described so far, is the incremental open-ended learning in artificial agents. Large corpuses of training data, predefined vocabularies and fixed mechanisms for *ad-hoc* scenarios are not feasible to develop robots that interact naturally with humans. The scope of our work is the runtime acquisition of concepts in an open-ended manner, from non-anticipated vocabularies. Relevant contributions with similar scope include research from human-robot interaction, which investigate learning by demonstration (Thomaz and Cakmak, 2009; Cakmak et al., 2010), with no language acquisition. Action planning in partially-complete robot's workspaces has been addressed through a probabilistic model that utilises background knowledge to fill the planning gaps with semantic co-associations learned from corpora of task descriptions (Nyga et al., 2018). Further studies use dialogue-based descriptions, in which the human interactively explains to the robot the meaning of a requested action, by breaking it down in simpler step-by-step verbal descriptions, to follow instructions that were previously unknown (Cantrell et al., 2011). A novel and cognitively plausible framework capable of learning grammar constructions has demonstrated how a robot can start with limited knowledge on the meaning of words and gradually acquire language-visual mappings to parse commands on previously unseen objects (Alomari et al., 2017). These works investigate only specific aspects regarding either vision, action or language learning. Instead, we study the integrated acquisition of language, language-percept, and language-motor representations at runtime and in unexplored scenes.

## Materials and Methods

### The Cognitive Model Framework

We draw our novel learning methodology using the brain-inspired framework, ANNABELL (Golosio et al., 2015), which is a large-scale neural architecture intended to help understand the level of cognitive development required for early language acquisition and development.

The framework is inspired by the Baddeley's WM model (Baddeley, 2012) and the Cowan's WM model (Cowan, 1998). These models are high-level functional systems of the working memory, which can be implemented either as symbolic or connectionist systems. Although natural language is considered to be a strong symbolic activity, information elaboration in our cognitive system follows the principle of neural processing over symbolic manipulation (Golosio et al., 2015). In the course of information processing, the activation patterns of the synaptic connections determine the appropriate paths of neural connectivity that guide the processing steps. Symbolic systems are unable to recognise regularities in large datasets. Such regularities in our architecture are handled by the comparison structure (section The Mental Action Sequence). Moreover, the model learns from only simple rules operating at a neural level, instead of detailed innate knowledge, which is compatible with the connectionist approach (McClelland and Kawamoto, 1986; Elman, 1991; Miikkulainen, 1993).

**Figure 1:**
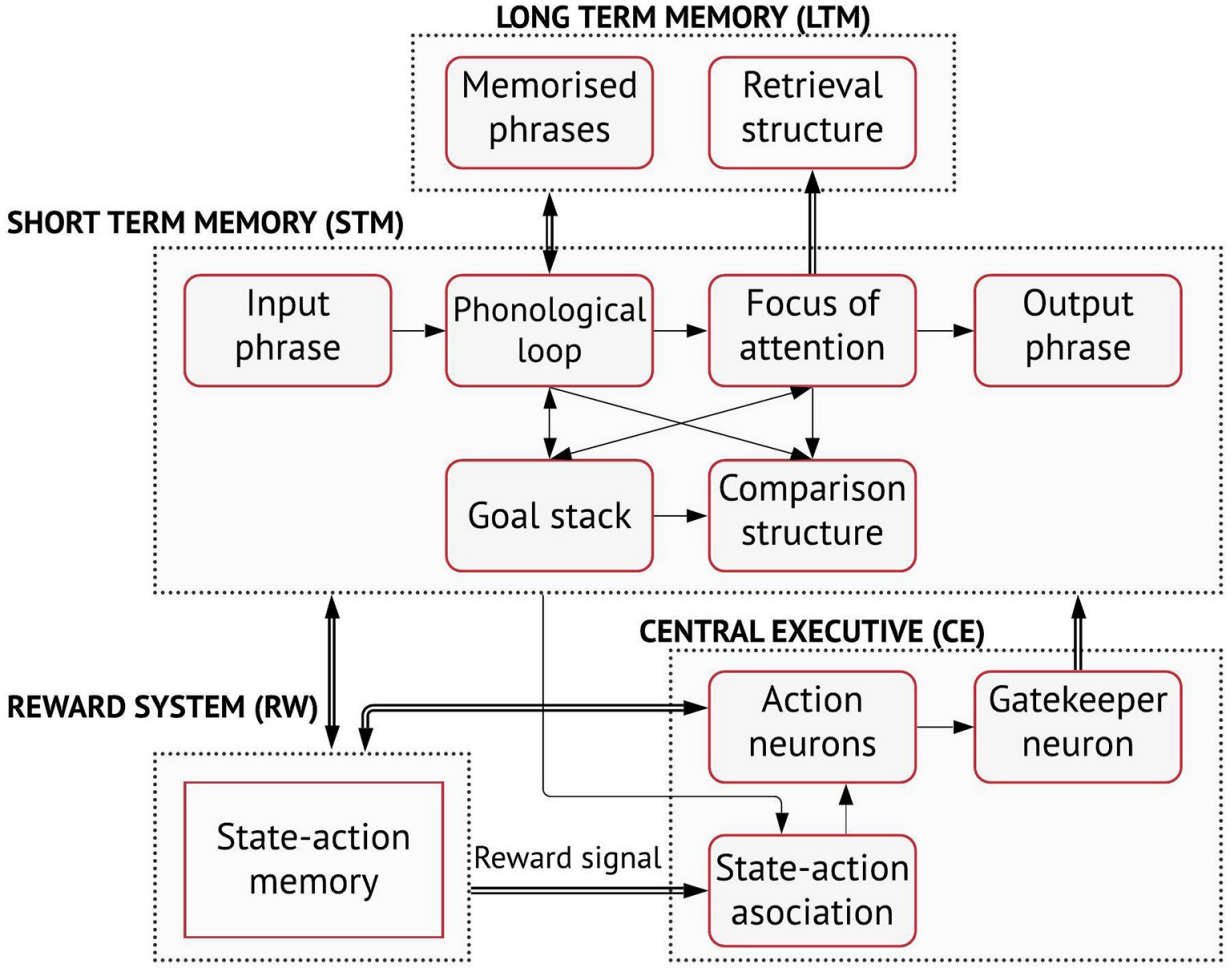
The cognitive architecture (courtesy of Golosio et al., 2015).

The global organisation of the model comprises four main neural components: a short-term memory (STM), a long-term memory (LTM), a central executive (CE) and a reward structure (RW) (Figure 1). The LTM includes a structure for phrase memorisation and another for phrase retrieval. The factual information stored permanently in the LTM comprises the declarative knowledge of the system acquired in some form during past experiences. The memorised phrases can be retrieved for task-solving using the focus of attention as cue. The focus of attention, in the STM, can hold up to four words. Other components of the STM include the phonological loop, which maintains the working phrase that can be either acquired in input or retrieved from the LTM, the goal stack, which stores goal chunks when an action that contributes to decision-making cannot be performed immediately and, a comparison structure. The comparison structure recognises similarities among words in the phonological loop, the focus of attention and the goal stack to further contribute with the decision-making processes.

A notable feature of the model is the use of a central executive for language processing tasks. The CE is a neural structure that controls all the statistical decision-dependent processes. It includes a state-action association (SAA) system, a set of action neurons and a set of gatekeeper neurons. These neurons are based on the same neural model, but specialise on the way they are connected to other sub-networks. The action neurons are used to trigger elementary operations, called *mental actions*, on word, word groups or phrases, for instance for acquiring words of the input phrase, memorising phrases, extracting words from the working phrase, retrieving memorised phrases from word groups, etc. Mental actions are performed when action neurons activate simultaneously one or more gatekeeper neurons. The connections between action and gatekeeper neurons have fixed, predetermined weights, so that each action neuron only corresponds to a specific operation. By activating the gatekeeper neurons, the action neurons control the flow of information among the slave-systems. The neural gating mechanism is instrumental in the cortex and other parts of the brain. The output connections of the gatekeeper neurons are generally fully connected to one or more sub-networks and can allow or inhibit the flow of signal through such sub-networks.

The state-action association (SAA) is a neural network that is trained to associate mental actions performed by the system to the internal states of the system. The SAA decides which action neurons are active that, in turn, control the gating mechanism. The state-action association is memorised by a reward system that triggers a synaptic change of the state-action association connections, when a target output is produced. One fundamental property that grants the generalisation capabilities of the system is that the *learnable connections*, which are the connections affected by the reward system, are connected to the action neurons, rather than being directly connected to output words or phrases. Therefore, by memorising the mental actions associated with words, word groups and phrases, rather than learning words or word combinations to solve certain tasks, the system learns preferentially to build the output. This allows to handle several tasks in input and, unlike classical state-of-art deep-learning models, to learn complex rules from only a few examples, similar to how humans naturally learn to perform tasks with reasonable accuracy from only a few instances. An extended description of the model and its connections to neuroscience can be found in the work of Golosio et al. (2015). The source code of the software, the User Guide of how to train and test the model, including all datasets used for validation are available at https://github.com/golosio/annabell/wiki.

#### The Mental Action Sequence

At this point, we explain why a neural architecture as opposed to a symbolic system is necessary to model the mental action sequence. The decision processes operated by the central executive are not rule-based but statistical decision processes. Let us consider how our model can solve the task “*add number 1 to the sequence of digits 2 5 7 8,”* which is a classical task used to study the working memory capacity. We simplify our discussion by assuming that the system has some initial knowledge on simple arithmetic additions, acquired from past experiences, to keep the cognitive load small. Therefore, declarative phrases like “*X plus Y equals Z”* are memorised in the LTM. The system is guided to perform the following sequence of elementary operations: (1) Transfer the phrase “add number one” to the phonological store and to the goal store. (2) Transfer the digits 2 5 7 8 to the phonological store. (3) Transfer the first digit (2) to the focus of attention. (4) Use this digit (2) to retrieve relevant information from the LTM, for instance “two plus one equals three” that is appropriate for the requested arithmetic operation. (5) Transfer the result (three) to the focus of attention and use it in sentence production. (6) Transfer the initial sequence of digits 2 5 7 8 to the phonological store. (7) Transfer the second digit (5) to the focus of attention. (8) Execute the mental actions until an output is produced (4-5) and repeat iteratively until the last digit of the sequence is processed. Let us assess how generalisation can arise in this example, when we test the system on a similar task using a different sequence: “*add number 4 to the sequence of digits 5 3 6 9.”* Since this sentence is similar to the learned task, the central executive will provide the same mental-action sequence. Actions 1-3 will be repeated, with the digit 5 in the focus of attention. The validity of the produced result depends on the retrieval process. Let us consider that relevant phrases in the LTM that contain number four are “*five plus four equals nine”* (a), “*five plus one equals six”* (b), where (b) is the most similar to the phrase retrieved during training (*two plus one equals three*). If the retrieval process is not modelled as a statistical process but depends on the similarity score only, a wrong sentence would be produced i.e., six instead of *nine*. However, the working phrase is also stored in the goal-stack (typical in cognitive architectures) until the end of the task. During decision-making, the comparison structure can recognise that phrase (a) and the phrase stored in the goal stack, both contain the number “*four.”* When the neurons of the comparison structure are activated, phrase (a) will be retrieved instead of (b) and the correct answer “*nine”* will be produced.

### The Integrated Robotic System

Duffy and Joue (2000) distinguish between a robot being only a controller with actuators and preceptors that performs in the environment without being part of it *per se* and the robot being part of that environment so that the direct interaction with the environment influences the robot's real-time learning, adaptation, and development in it. They define the former as ON-World or *weak* embodiment and the latter as IN-World or *strong* embodiment. These notions are also supported in the work of Sharkey and Zeimke (2000). In this work, the robot is placed in the physical world and functions autonomously by means of appropriate elaboration inside a cognitive model of the sensory inputs that situate the body of the robot in its internal map. In this sense, the robot is “ON” its environment. However, in the next sections, we will demonstrate how we start from an initial set of motor primitives to generate new maps and representations of the world to interact with and influence the environment beyond the static internal representation of the robot.

**Figure 2:**
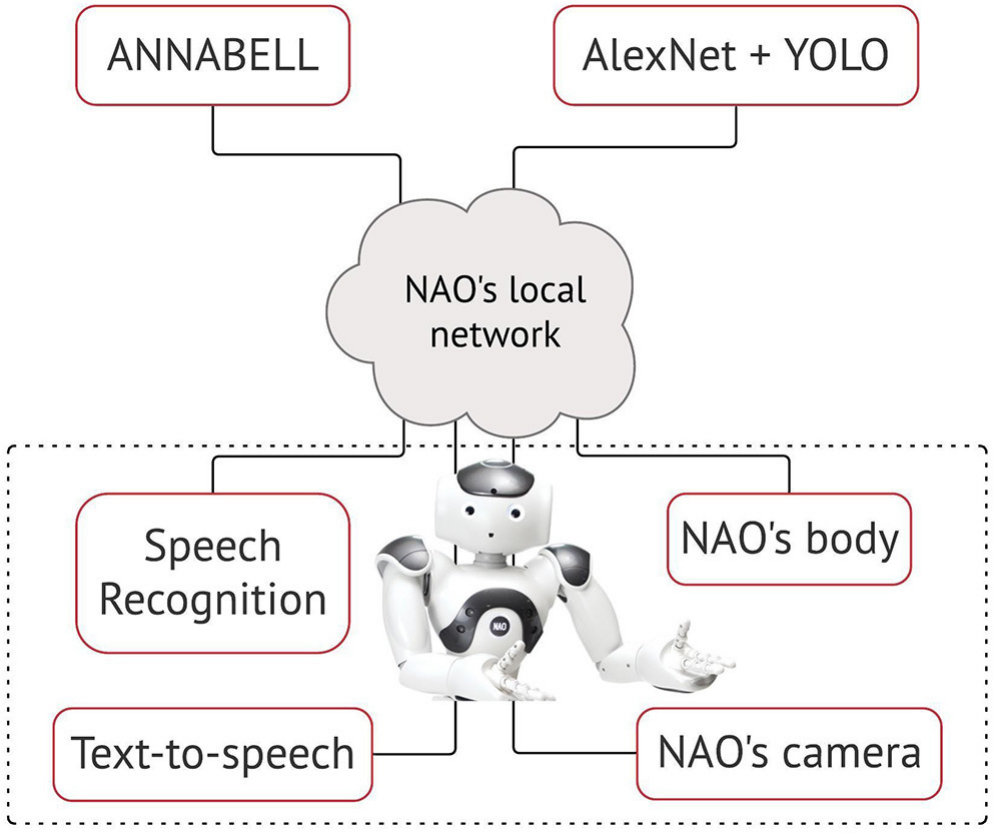
The on-world embodiment of the cognitive model ANNABELL on the NAO robot.

We selected a humanoid robot, following the intuitive reasoning that a physical body is necessary to act in the environment and, that natural language as a communication interface is arguably a human attribute. In this work, we used the academic edition of NAO robot, version 6 (SoftBank Robotics, 2021). We implemented two pre-trained state-of-art vision recognition systems, namely Alexnet/Wordnet (Krizhevsky et al., 2012) for object classification and YOLO (Redmon et al., 2016) for object tracking, using Tensorflow2.0 and Keras. We preferred AlexNet over its latest higher-accuracy competitors, because of its faster training time and lower computational power requirement. It renders an acceptable classification performance for the object classes used in our perception learning experiments. We switched to the YOLO model for the action learning experiments to grant fast real-time tracking of objects and improve sensory inaccuracies. Our aim was to maintain the external components lightweight and allocate the available computing resources to the training and validation of the large-scale cognitive model. Given that our architecture and methodology are independent of the coupled modules, the final system can be greatly optimised with more advanced deep vision or robotic frameworks. Indeed, NAO's motor skills are limited, however its relatively low cost and computational effort make it suitable for the type of experiments performed here, which place a greater emphasis on the cognitive model rather than the robot actuator. We preserved the NAO's speech recognition module but enhanced its vision system with the deep models explained above. The NAO's cameras capture real-time frames and redirect them to the detection/tracking systems. The auditory, visual and the motor outputs (explained in section The Proposed Learning Methodology) are sent over to the cognitive model which elaborates the multimodal information. We interfaced the cognitive model, the NAO robot and the smart vision via a client-server protocol. In the server side, the model runs in a remote computer and communicates in two directions with the robot via the robot's local network (Figure 2).

## The Proposed Learning Methodology

In the described framework, there does not exist a visual neural component to elaborate sub-symbolic data. To overcome this limitation, without increasing the complexity of the model, here we propose a method of representing sub-symbolic information, from the physical world, in symbolic form, in the model's phonological loop. According to Baddeley (2012), auditory verbal information and visually presented language can be transformed into phonological code and encoded in the phonological store. We exploit this claim in our technique to include auditory and visual stimuli from real workspaces in the model and elaborate it similarly to verbal language, granting appropriate disambiguation so that neither of the (symbolic and sub-symbolic) processing affects the efficacy of the other. In this light, our model implementation complies best with the Cowan's WM theoretical model, which suggests the presence of an “activated memory” as a subset of the long-term memory (LTM). The activated memory can hold a large number of activated elements. In particular, it comprises the focus of attention, which, according to Baddeley (2012), has a similar role to the episodic buffer of the Baddeley's model, designed to bind domain-specific information (phonological, visual, semantic) in integrated units of information with chronological sequence. Similarly, by using our novel convention of the internal representation of the information from different domains (linguistic and non-linguistic), we are able to process multimodal information in the same subset of the memory.

In our method, for every word (semantic form) that has a direct mapping in the physical environment (meaning), we construct its corresponding sub-symbolic representation (percept or motor stimuli) by prepending an underscore “_” to the word, to yield the pair <_word word>, where <_*word*> is the visual/sensory component and < *word*> is the language component, as seen by the model. Notice that this is only a convention technique used in the cognitive model for information elaboration purposes, to disambiguate in sentence level between sensorial and linguistic information. Here, < *_word*> is a symbolic internal representation of the robot's vision and motor system. Hence, it encodes a real binary stream representation of an image or a motor trajectory, i.e., the sequence of joint angle vectors for robot body movements along predefined trajectories (section The Robot Learning Experiments), in the phonological store of the model.

Encoding sub-symbolic information in symbolic form (_*word)* as opposed to bit stream representations, any dataset labels (e.g., image net classes, n02119789) or joint angle vectors allows to:

- Parse and elaborate visually presented data similarly to verbal inputs and process both simultaneously, while properly disambiguating the information contained from sensory and that from language; *therefore, our methodology is applicable in other potential complex cognitive architectures that address language acquisition through embodiment, regardless of the larger integrated system*.
- Generate a <form, meaning> pair for every new word (limited with fixed dataset labels) and a unique representation even when image bit streams differ for the same semantic (e.g., different images of the same object); *scalability and open-ended learning*.
- Grant such knowledge representation in the model and appropriate representation and retrieval mechanisms, so that the multimodal information elaboration is a pure attribute of the cognitive architecture, making it independent of the external coupled modules or robotic platforms; *optimisation*.

Each sub-symbolic element is represented as a <*_word*> symbol. These elements can indicate a main classification, optional adjectives (“big,” “red”) or the position or the visual element in a scene (“left,” “right,” “centre”). A scene is represented by a sequence of <*_word word*> mappings. In the acquisition stage, these pairs are stored in the long-term memory (LTM) jointly with the linguistic information and are elaborated in the same phonological loop after retrieval.

**Figure 3:**
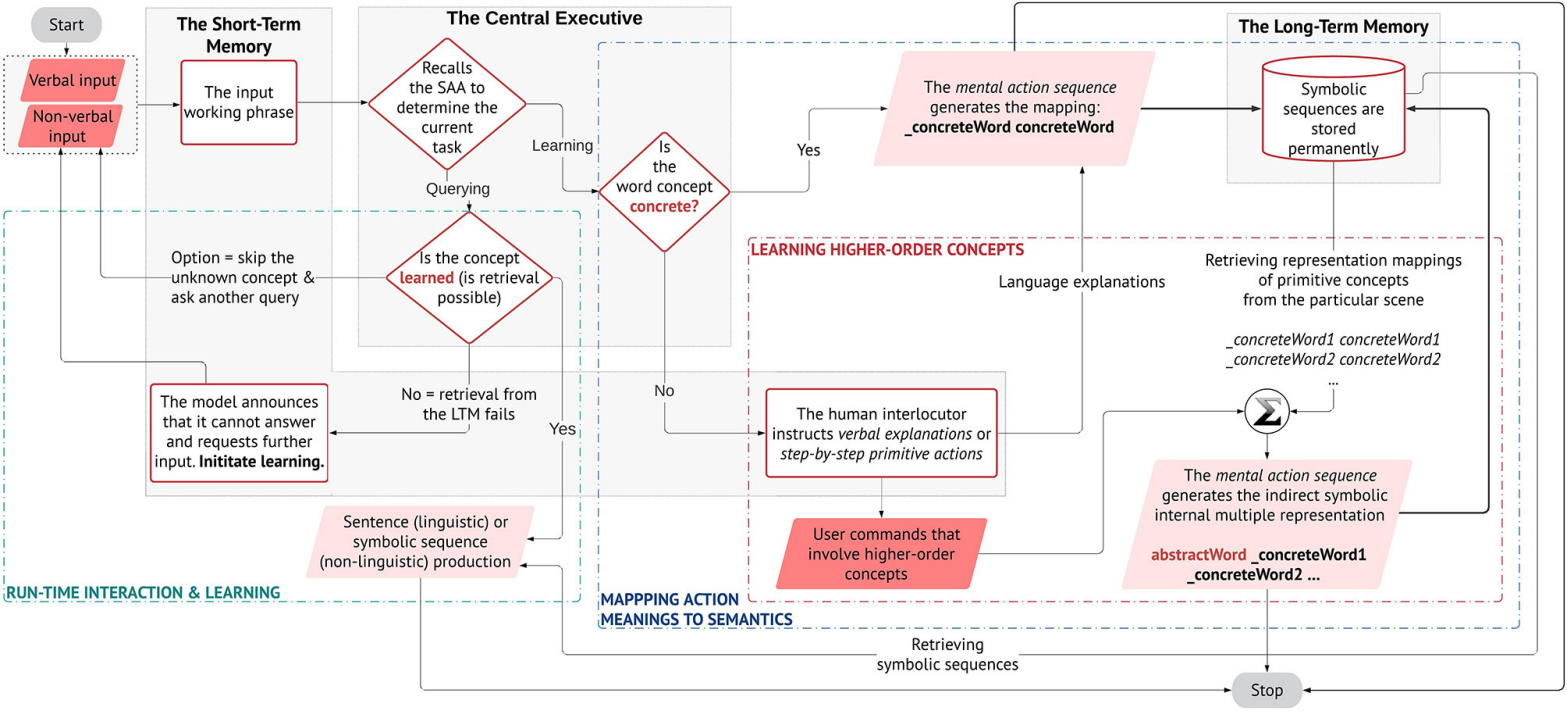
The proposed learning methodology of meanings and language constructions. The central executive (CE), the long-term memory (LTM), and the short-term memory (STM) of the architecture are shown. The CE handles the decision-dependent processes, but not in a conventional if-else logic; rather, decisions are made based on the state-action association that is learned, memorised, and rewarded during the initial training. As a result, it can disambiguate the type of task that is handled by the STM and call the respective mental action sequence. The STM elaborates the working phrases through the mental actions. The LTM stores the symbolic sequences and is accessed during phrase retrieval to produce a valid output. The parallelograms represent either the user's input (dark) or the system's output (light). The output can be a spoken sentence or a symbolic phrase (actions to be executed by the robot). The interlocutor decides preferentially at run-time to teach a new concept (object/action) or to query the model (e.g., to learn an object or to perform an action).

In Figure 3, we illustrate the proposed learning methodology that leads to our contributions. From the diagram, it can be seen how we address our aim: the pairing of verbal semantics with their meaning in the physical environment and, the acquisition of higher-order concepts by transferring the meaning of primitive concepts guided by natural language utterances, in an interactive open-ended manner at run-time. Differently from previous studies (Golosio et al., 2015; Giorgi et al., 2020) whither the authors demonstrate the system's ability in sentence processing and production, here we investigate how the model can elaborate natural language received from auditory stimulus, in interaction with non-verbal stimuli from vision and the physical body, to acquire and partially reproduce linguistic concepts in its workspace for problem-solving purposes. Our target is vocabulary acquisition of linguistic concepts as a natural consequence of the agent's behavioural praxis and interactions in its situated scenes.

The different neural components of the architecture are shown in the methodology flowchart given their specific roles (Figure 3). The central executive (CE) manages the decision-dependent processes. When a phrase arrives in input, the CE recalls the state-action association that has been memorised and rewarded during the initial training, to determine the type of task and produce the respective mental action sequence. The short-term memory (STM) handles all the mental actions from word/phrase extraction, to phrase retrieval and sentence production.

### Learning to Map Primitive and Higher-Order Concepts in the Workspace

If the task is learning, the CE further decides whether the concept that is being learned is concrete or abstract (on user's input). We model learning, respectively, for primitive or higher-order concepts:

1. Primitive concepts are directly represented as <*_word word*> pairs (concreteWord in Figure 3 is only used for convention), where *_word* represents the sub-symbol (meaning) and *word* its respective semantic (form). These pairs are stored permanently in the long-term memory (*_concreteWord concreteWord)*.
2. Higher-order concepts are indirectly hooked in multiple representations (language and actions). The user gives verbal explanations or systematic guidance using primitive actions, which describe the abstract concept, e.g., *concreteWord1, concreteWord2*, etc. These are learned as in (1) and stored in the LTM. For the abstract concepts, the STM produces a sequence by retrieving from the memory and linking the primitive actions that are associated with the abstract concept *(abstractWord_concreteWord1_concreteWord2 …)*. Notice how *abstractWord* does not have a sub-symbolic representation, i.e., *_abstractWord*, as it cannot be directly mapped in the physical workspace.

### Run-Time Interactive Learning

After the initial pre-training, the interlocutor can choose to query or continue teaching new concepts. Again, the CE decides which task is the user initiating. The model can only answer if it has learned a concept (as explained above). If some concept is not learned, but is queried, the CE infers that a phrase retrieval is not possible to produce a valid output. Therefore, it will announce the inconsistency to the user. On the user's decision, the CE will initiate learning (teach the concept) or querying (skip and ask a new query). The interactive learning process is not interrupted and the model can continue to learn new instances. The flexible learning mechanisms and knowledge representation in the LTM allow the system to learn an unrestricted number of new concepts and apply the newly acquired data directly on similar tasks, without training the whole model anew.

## The Robot Learning Experiments

We apply and test our methodology in verification experiments with the final robotic system, which can be regarded as:

1. *Perception tasks*, where the model learns objects in its surroundings, maps object semantics to their corresponding percept and engages in simple gameplay with the human on these objects;
2. *Manipulation tasks*, where the model learns to act on assorted objects in its workspace, maps concrete action semantics to motor actuators and transfers the meaning of primitive actions to learn higher-order (abstract) semantics, while engaging in manipulation tasks.

To execute either tasks, the system must know or learn the <_object object> and/or the <_action action> pairs, where _*object* is the object seen and used by the robot and _*action* is the basic action perceived and acted by the robot. We first explain the proposed methodology and designed scenarios of training the system to learn the <_word word> mappings, which are memorised in the long-term memory and retrieved during task solving. Afterwards, we demonstrate how these mappings are acquired at run-time and used across similar tasks to generalise when the workspace or language instructions change, to learn new objects and actions, in non-anticipated scenes. In the following (sub)sections we describe this separately for perception and action tasks.

### Perception Tasks

In this type of experiments, we engage in a simple gameplay with the embodied system, in which the robot is asked to recognise some animals and their member categories (mammals, reptiles,…). The only action performed here by the robot is pointing. There are three elementary tasks:

1. The robot is asked to tell the name and category of an animal it sees.
2. The robot is asked to determine which between the two animals it sees belongs to a specific category.
3. The human and robot play a game, in which given three random animal names, the human shows only two animals to the robot and the robot identifies which animal is missing.

#### Target Training Data

In the preliminary training, the model has only stored in the long-term memory (LTM) the <_dog dog>, <_frog frog> and <_snake snake> pairs. It has also memorised declarative phrases, such as “the dog is a mammal,” “the frog is an amphibian” and “the snake is a reptile.” This declarative knowledge is used to train the model the appropriate mental actions to answer to the user queries (Tasks 1-3). The input working phrases that the model elaborates are constructed by appending the information from the vision system (e.g., _*dog*) to the spoken language transcripts from speech recognition (e.g., *what is this*). We explain more in-depth how this input phrase is obtained later in this section.

To train Task 1, we used the following queries:


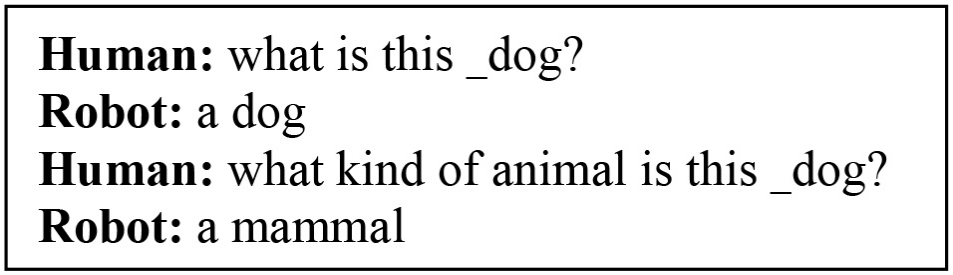


Suppose that, in Task 1, we want to query the system on an animal it has never seen before, e.g., *elephant*. Given that an <_elephant elephant> pair is not yet found in the long-term memory, a retrieval process cannot occur. When the retrieval fails, the system is trained to announce that it does not know the answer. Therefore, to the queries, “*what is this _elephant?”* or “*what kind of animal is this _elephant”* the system will initially answer “*I don't know this.”* From a human perspective, if we are told the correct answer, we should be able to know it when queried again. Therefore, we further train the model to use the auxiliary response from the human, to build a valid answer for the query that was previously unresolved. In this case, the user dictates that “*This is an elephant”* and “*The elephant is a mammal.”* When re-queried “*What is this _elephant?”* or “*What kind of animal is this _elephant?,”* the system self-amends its past reply “I don't know” to “*an elephant”* and “*a mammal,”* respectively. This type of learning using a language feedback has also been studied by Twiefel et al. (2016) and is particularly useful in real-world human-robot interactions. The authors propose a rule-based inference module which can identify cases of inconsistency in the incoming sensorial information from the current workspace configuration, reject invalid inputs and maintain a feedback loop to the user to change the uttered instruction. In this work, the inferences on inconsistent information are handled by the central executive, which decides whether a retrieval step in the mental action sequence is not possible and then recalls the appropriate state-action association sequence to initiate the feedback. The feedback applied here is used merely for the architecture to self-infer which information it already knows about its physical world or the task at hand.

In Task 2, we train the model the following:


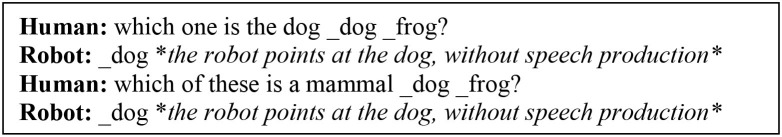


In Task 3, the model is trained to find the missing animal:


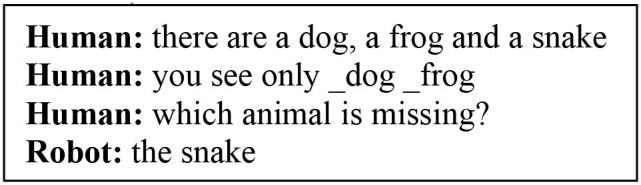


It can be seen how, for this context only, the robot “interprets” the inferred concept of a *missing* object as something that is not visually seen in his perceived workspace.

#### Object Learning

The purpose of perception tasks is not animal classification, which can be done with any object recognition or neural network classifier. This study aims to demonstrate that the model can acquire new semantic meanings at runtime, self-map them in the physical environment and use these newly acquired mappings, to generalise on novel tasks, similar to those trained but using new data. The model should apply this information autonomously, without being re-trained on the entire scale-up corpus, granting a degree of adaptability in unknown workspaces.

**Figure 4:**
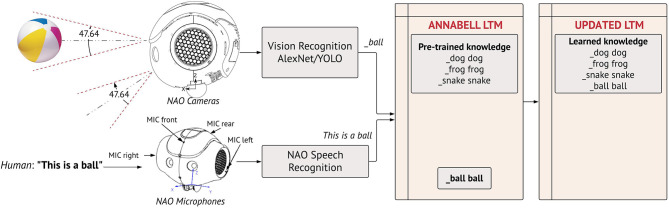
Object learning. The robot is shown an object (ball) and the human dictates what the object is (This is a ball). The visual recognition system produces a label for the classified object. This label, merged with the phrase detected from NAO's speech recognition module, are used in the cognitive model to generate the pair <_ball ball> that is stored permanently in the long-term memory (LTM). The LTM is autonomously amended to include the novel element as a learned information, which can be appropriately recalled during the mental action sequence execution. This setup is used to collect the target test data, by showing to NAO printed images of animals for Perception Tasks and physical objects in Manipulation Tasks.

We demonstrate how the robot learns new objects in its workspace, not included in the initial target training data, with the help of human guidance. In Figure 4, we illustrate this run-time learning process with the object *ball*. Similar to a natural communication, during the live experiment, the human shows the object to the robot and dictates what the object is. The object instance captured by NAO's cameras is processed by the object detector, which converts it to the symbol *_ball*. The spoken utterance is converted to transcript by the speech module. Both are appended at the input of the cognitive model (*This is a ball _ball*). The architecture self-generates a <_ball ball> pair that is stored permanently in the long-term memory. At this point one may ask why the model extracts the word *ball* and ignores the function words in the phrase (this, is, a). The symbol generated by the object detector correlates best with *ball* among the other words of the phrase and is, thereby, extracted to build the pair (comparison structure, Figure 1). As a result, only perceived objects are directly mapped and memorised.

When an object is shown to the robot and a description is uttered by the human, a <_word word> pair is generated and stored in the long-term memory for that object (Figure 4). Each time the image is re-used, the recognition system will generate the same *_word* representation. Upon receiving this as input, the central executive can identify that this object is represented symbolically in the LTM with the pair <_word word>. If a new object is shown to the robot, with no utterance on its semantic meaning, the recognition system will automatically represent it in symbolic form, say *_word1*. However, there exists not a <_word1 word1> pair in the LTM, hence the model will not recognise the object (the example of *elephant* explained in Target Training Data).

#### Target Test Data

The pre-training data used as the taxonomic knowledge to learn tasks 1-3 include only three animals (*dog, frog, snake*) and their respective declarative sentences (section Target Training Data). To assess the generalisation skill of the model, the target test data are acquired gradually at run-time, following the method of object learning (section Object Learning). In the test, it is verified if the agent can continue to scale-up the knowledge stored in the long-term memory, without altering what has been acquired previously, its ability to inhere whether the current knowledge is consistent to solve the task and, the generalisation skill on similar tasks, with the newly acquired data.

The robot is taught the new objects directly from verbal utterances and live captured images. The setup is illustrated in Figure 4. The test data are collected as follows. The NAO robot, the speech and object recognition modules operate on the client side and communicate bi-directionally with the architecture on the server end. The human shows NAO a new animal by holding an image of the animal in front of the robot. NAO's cameras feed the object recognition module with the image instances captured in the robot's visibility area, which categorises the objects directly from the visual information obtained by the cameras. The output of the object recognition module is the corresponding classification label, which is stored symbolically in a string (*_word*). The human dictates the animal. The auditory signal is captured by the robot's microphone and is processed by the speech module, converted to text and stored in a separate string (linguistic form, *word*). The strings are appended and sent over to the cognitive model. Both the input and output of the cognitive model are loosely monitored by the human observer, to pre-filter sensory-based errors in the samples and to ensure that the correct pairs are learned (section Handling Sensor-Based Uncertainty). Otherwise, the human proceeds with a new animal. When sufficient animals are learned, we query the system on the newly collected data using natural spoken language and images. We repeat the tasks 1–3, using similar interrogative phrases, but showing the robot the new animals of choice. For *Perception Tasks*, we used printed images of animals. In *Manipulation Tasks*, we used real objects to populate the long-term memory with <_word word> pairs of the learned objects.

In subsection Target Training Data, we explained what would happen if the system were queried on an animal (Task 1—test stage), before learning the animal, using the example of *elephant*. In the cases of inconsistency, the model has learned to respond with “*I do not know.”* The human might choose to repeat the learning process explained above, to teach the animal to the robot. The model will generate the appropriate pair and store it in the LTM. An important aspect and the target of this scenario, is that after updating the memory with the new mapping, the model will not answer “*I do not know,”* if the human re-queries on that animal. Instead, it will give the correct answer. The runtime test data acquisition determines if tasks 1–3 can be solved appropriately.

### Manipulation Tasks

In this type of experiments, we teach the robot to solve tasks using natural language instructions. To do so, the robot must map natural language to its internal motor map and, recognise the workspace and the task. We attempt to model the learning of higher-order actions in robots expressed through abstract concepts of natural language. This is achieved by teaching the agent step-by-step elementary actions, which it executes and then combines sequentially in some form to complete a high-level instruction. We use the <_action action> convention to map primitive actions (meaning) with their semantic, e.g., <_grasp grasp>. By combining natural language (from the human) and transferring the meaning of primitive actions (internal motor representation), we teach the robot the meanings conveyed by abstract notions of language, such as “take, use, make, ….”

#### Target Training Data

In the training stage, we teach the robot “how to make tea,” using a mug, water and a teabag. We start with an initial pre-training, during which the model has first learned the set of tools it will use, permanently memorised as <_mug mug>, <_bottle bottle> and <_teabag teabag> (as in Perception Tasks). The objects are placed on a table-top setting; therefore, the *table* is part of the workspace. Moreover, we have pre-trained the mappings between primitive action semantics and internal motor representations of those actions, memorised in the LTM as <_grasp grasp>, <_lift lift> and so on. The setup is as follows. Initially, we pre-defined the action primitives on the robot. We ran NAO in record modality (enabled on a real robot) to train it the set of primitive actions via direct motor exploration. The joint movement are regulated to produce trajectory formation for primitive actions. We encode primitive actions in symbolic form, where each symbol now represents the sequence of joint angle vectors along the predefined trajectories (e.g., *_grasp* is mapped to a respective grasping-like trajectory in the internal motor map of the robot). Although, we pre-select the series of motor primitives, we enable the model to self-learn the mappings between the action and the internal representation corresponding to the action, by means of the trained mental actions. Moreover, when learning abstract actions, for which there are not pre-defined trajectories, though we exploit a static internal motor representation of the robot, we are able to generate new representations and motor maps, to produce novel robot behaviours in the environment. Hence, the robot can adapt at some extent and execute tasks that are not pre-determined in its internal system, in new scenes.

**Figure 5:**
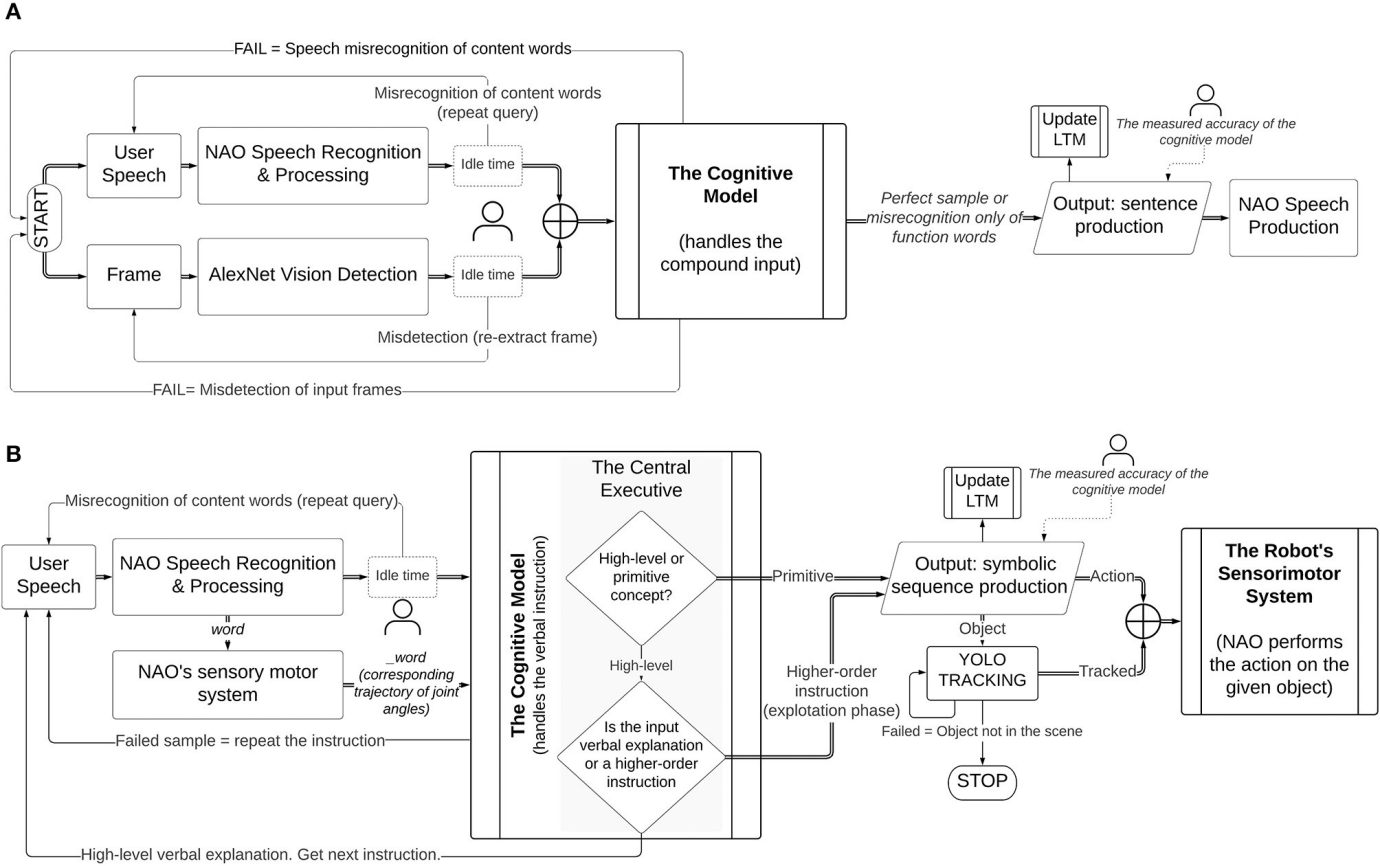
Handling sensory-based inaccuracies during the runtime acquisition of target test data. In Perception Tasks **(A)**, the model takes the compound input from user speech, which is processed by NAO's speech recognition module and, a vision input, captured by NAO's cameras and classified by AlexNet. The human tutor loosely filters potential sensory errors to ensure ground-truth sample inputs at the cognitive model. In the event of a speech (content words) or vision misrecognition, the human re-captures the input. The accuracy is measured at the ends of the cognitive architecture only and is defined as the correctness of the output, assuming error-free input samples. Similarly, in Manipulation Tasks **(B)**, the cognitive model takes a pre-filtered compound input of speech samples and trajectory information from the robot's motor map. The motor information is self-extracted from the robot and mapped to its symbolic representation without human supervision. This is fundamental for the test acquisition toolchain from primitive to high-level actions.

In Figure 5B, it is illustrated how the cognitive architecture learns the mappings between the primitive action and the internal representation corresponding to the action. When the human chooses to teach a new action, the robot self-extracts the corresponding trajectory from its internal motor system, encoded in symbolic form. This is combined with the spoken utterance of the action and is processed in the cognitive model, to generate the <action, meaning> pair. We preferred using a mediating convention (*symbolic* representation) mapping technique instead of multidimensional vectors, to train the long and short-term memory units of our architecture the acquisition of a scale-up vocabulary of concepts. When the mappings for primitive actions are learned, they can be retrieved to solve further tasks, at a higher level of abstraction, as explained further in this section.

The basic and high-level instructions of making tea are specified by the tutor and are acquired gradually during the interaction. Here, we describe how concepts are learned and represented in the overall knowledge of the system. First, the human explains using natural language what high-level concept is achieved when executing one or more primitive actions. Let us consider the interaction below:


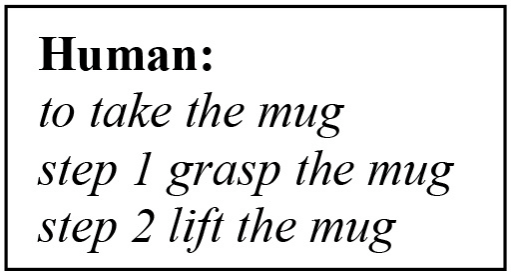


The first phrase is the verbal explanation of what will be the outcome of the high-level concept “*to take something.,”* in this context. The following 2-step actions are the physical exploration of the concept, which are defined in the robot's motor system. The model is trained to convert the basic instructions of type “step X do something,” into a symbolic sequence of actions and objects, e.g., *_grasp _mug* and *_lift _mug*, meaning that a grasp (lift) action will be carried on the object *mug*. These symbols create and maintain a bi-directional link to their sub-symbolic representatives. In other words, they are generated from sensory data and can retrieve sensory data (e.g., a trajectory). Thus, a symbolic sequence generated by the architecture is the symbolic internal representation of the robot's motor system.

After the primitive actions 1-2 have been executed, the human instructs “Nao take the mug.” The model has been trained to recall and combine the two primitive steps, linked to the action *take*. In other words, for this instruction in input, our model will build the mental action sequence that generates the symbolic sequence <take _mug _grasp _lift> and the state-action association that produced this output is rewarded and permanently memorised. Notice how *take* does not have a symbolic link to the robot's motor system, because its trajectory is the result of combining the pre-defined trajectories of *grasp* and *lift* on the percept *mug*. We name this a high-level symbolic sequence, to identify that it relates to an abstract concept.

The interlocutor can flexibly add other instructions, by providing a verbal explanation and a step.


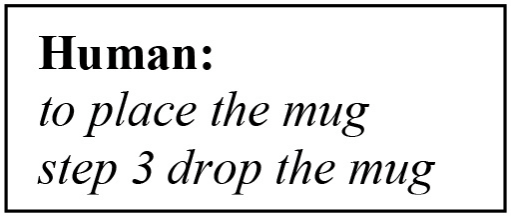


**Figure 6:**
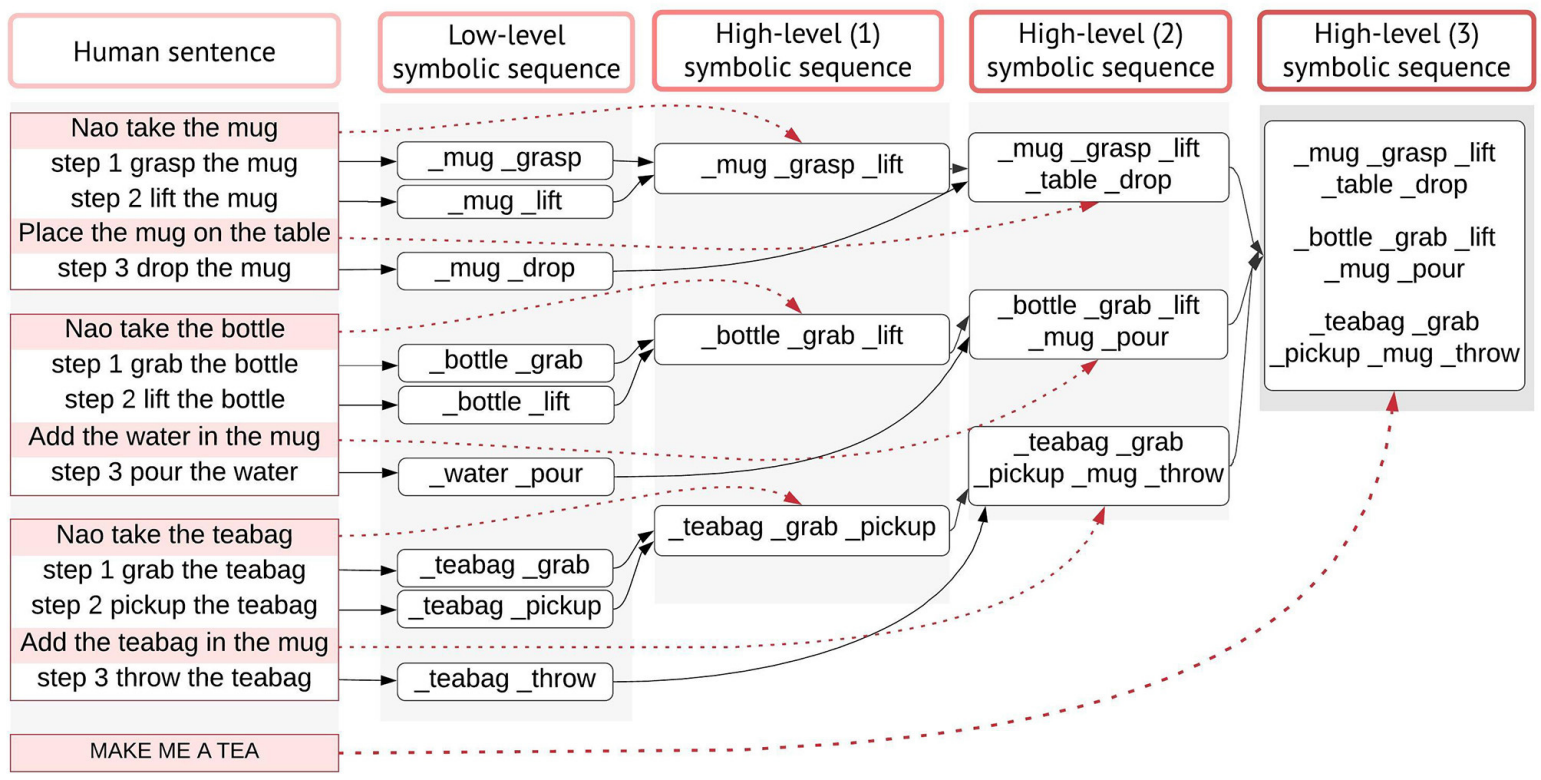
Action learning. Primitive action semantics (i.e., concrete actions) are mapped directly to their internal motor representation and the <_action action> pairs are stored permanently in the long-term memory (LTM). For each instruction of type “step 1 do something,” the model produces a low-level symbolic sequence, by extracting the representations that have a <_word word> mapping in the LTM. For higher-order actions that do not have a direct symbolic representation of the action (e.g., take), the model retrieves and links the primitive action meanings that are instructed sequentially by the human. First high-level sequences are built by transferring the meaning of 2-step primitive actions. Second high-level sequences link the meanings from the predecessor level with the subsequent primitive action. Third high-level sequences are achieved by recalling the second high-level sequences. High-level symbolic sequences correspond to the representation of abstract notions (take, use, make) in lower-order non-linguistic primitives. The dotted lines indicate how high-level utterances are internally represented indirectly by associating lower-order representations. Notice how same abstract concepts (e.g., take) have different symbolic internal representations, depending on the outcome/tools.

In Figure 6, we illustrate the process of generating higher-order actions from linking primitive actions in sequential order, or with other high-level symbolic sequences. The main task in this scenario is to make tea, given the verbal explanation “to make tea you need a mug, water and a teabag.” The model will first attempt to perform basic operations with the other objects (bottle, teabag), by reproducing the mental sequence as explained above, illustrated in Figure 6. Eventually, the model will generate all necessary low/high-level sequences and link them together to execute the task.

**Figure 7:**
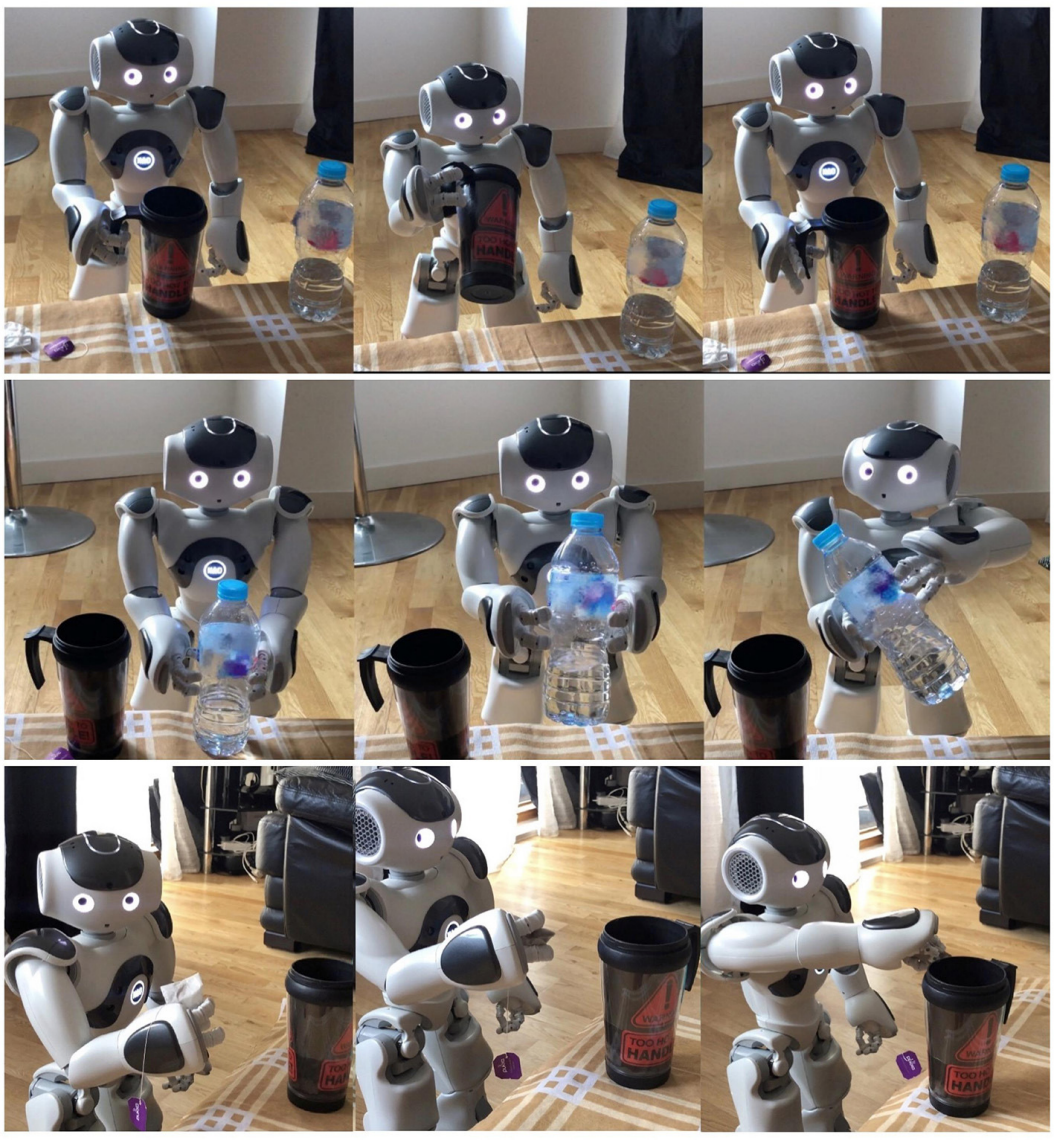
Demonstration of the NAO robot performing the task “NAO make me a tea.”

#### Action Learning

In *perception tasks*, we explained how the system learns new objects at run-time. Therefore, to solve new tasks in new workspaces, the robot is first taught the set of novel objects/tools as in object learning (section Object Learning). In this section, we explain how the system learns action mappings at run-time. Actions acquired through natural language instructions must be properly represented in the long-term memory so that this knowledge can be retrieved in novel endeavours. They must also be appropriately linked in the robot's motor system. The primitive actions are initially implemented in the robot using predefined trajectories. The integrated model can autonomously map the internal knowledge representation of the cognitive model with the respective internal motor representation of the robot.

Here, we explain how to populate the long-term memory with auxiliary primitive actions paired with their meaning extracted from the motor system (Figure 5). To train the CE to identify the action being primitive or high-order, we used distinct spoken phrases. Single-word instructions identify primitive actions. In this case, the auditory signal is used both in NAO's speech module and to retrieve the respective trajectory of the action from the robot's internal motor map, symbolically represented. The linguistic and non-linguistic data are combined before entering the cognitive model and the <_action action> pair is permanently stored in the LTM. Similarly, pairs for other actions will populate the memory. Notice that an action is learned by the model only if it is mapped appropriately to its symbolic internal representation, i.e., an <_action action> pair is stored in the LTM. These can then be retrieved to execute primitive or higher-order instructions on-demand. The aim of the runtime acquisition of action semantics is for the robot to map novel semantics to their meaning in terms of robot's motor self-skills. Therefore, it is assumed that the robot already has a set of pre-defined skills in its internal map, allowing it to follow instructions from natural language. Similarly, Hinaut et al. (2014) explored the acquisition and production of grammatical constructions. In their method for action performing task, the meaning of a random action is obtained by having the robot generate the action using some objects in the workspace. The human observer generates a sentence that can be used to command the robot to perform that action. Thereby, <sentence, meaning> pairs are created to populate the database and are used during human-robot interaction to instruct the robot to perform actions. One major difference with our work is that, here, we do not observe the robot's behaviour and then construct a sentence that can command that behaviour. Instead, we first train the robot to map the instructions it hears with the primitive actions in its internal map and, afterwards, autonomously generate meanings for high-level sentences by combining its self-representations on primitive actions. The robot does not have a pre-defined representation for abstract concepts. Instead, their meaning is indirectly mapped to the meaning of its constituting primitive actions, provided by the human's step-by-step guidance. Semantic meanings are transferred from low level (primitive) to higher-order (abstract) as opposed to mapping higher-order concepts directly to a pre-defined motor exploration (Figure 6). Hence, the robot learns and represents new meanings for abstract actions in its internal system.

When the human requests an action, the architecture generates a symbolic sequence that commands the robot to execute the action on the relevant object. In the right-hand end (Figure 5B), an action trajectory can be retrieved only if both arguments are known, the *_action* and the *_object*. These arguments are self-extracted from the symbolic sequences outputted by the cognitive model. The *_object* first initiates an object tracking in YOLO using the robot's cameras and, when the match is found (object present in the workspace), *_object* is used as the second argument to complete the call of the action trajectory. Thereby, when the output action from the cognitive model matches the action primitive AND the output object matches the YOLO detection, the correct execution block is triggered in the robot. We implemented a waiting time on the robot to execute sequential instructions generated by the cognitive model or to expect further instructions from the human.

#### Target Test Data

To assess the model's generalisation capabilities, the robot is placed in new workspaces and/or is given instructions that involve different tools/objects and different ways to manipulate those objects, to obtain a certain outcome. There is no limitation in the number and type of novel objects that can be learned, however the test data acquisition has two main constrains. A set of primitive action trajectories must be initially predetermined in the robot, to ensure that action semantics are mapped to the robot's respective motor representation. Second, the types of phrases used to request (any) instructions must be similar to those trained, follow the same step sequence (1, 2, 3), and the final task must use exactly three objects with three manipulation steps for each object.

The test data is collected as follows. First, we teach the robot new objects, as explained in section Object Learning. The objects used here are real-world objects instead of images (Figure 6). After learning the objects, we dictate all the new primitive actions that are going to be used during the task (predesigned only at the robot). The integrated model will generate all the mappings, as explained in action learning and, memorise them permanently (Figure 5). Then, we begin the step-by-step guided operations that explain to the robot how to compose the higher-order concepts from primitive actions (forward direction). The instruction phrases should resemble the following pattern:


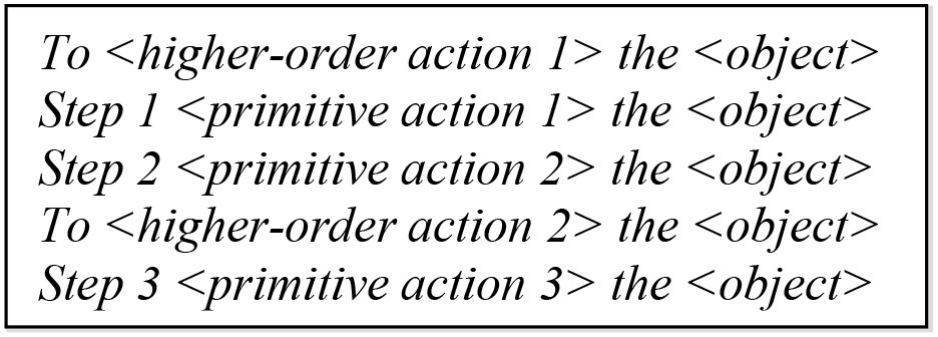


The Central Executive can disambiguate these phrases autonomously to determine if they infer a verbal explanation or an instruction. In the former case, the cognitive model does not start an exploitation phase, but waits for the next instruction. In the latter case, an exploitation phase is triggered, and the cognitive model generates a symbolic sequence (Figure 5). After the steps are learned, we request high-level instructions, similar to those in Figure 6 (column 1, human sentences, highlighted), but using the new objects for the given task. For example, if the model has been taught two steps to take the bottle, “step 1 grab the bottle, step 2 lift the bottle,” a high-level instruction would be “Nao take the bottle.” For this instruction, the model will decompose (backwards direction) the higher-order concept (*take*) into the respective primitive concepts. It is important to notice that, only for this scenario the outcome of the command *take* will be to “grab and lift,” because the object of reference is *bottle*, which determines what step-by-step lower-lever actions are recalled. In Figure 6, is demonstrated the NAO robot completing the task of making tea.

## Handling Sensor-Based Uncertainty

Figure 5 illustrates how the whole integrated model handles sensory inaccuracies of speech and vision recognition and, what is intended as the accuracy of the cognitive framework only (section Results).

In the *perception tasks* runtime tests (Figure 5A), the model takes two sensory inputs, either if the task is object learning (section Object Learning) or querying. The user speech transcript that is processed by NAO's speech module and the classification output of AlexNet are saved in separate strings. To obtain acceptable input samples closer to the ground truth, we pre-filter potential sensory errors by implementing a small idle time at the end of the speech and vision systems. This is done solely to enhance hardware limitations but does not affect the interaction from a real-world perspective. The sensory outputs are monitored by the human supervisor. If there is a speech misrecognition or object detection error, the human repeats the spoken command or shows the image again. The respective output strings are overwritten automatically. At the end of the idle time, the compound input is generated by appending the contents of both strings (a spoken phrase and a detected frame). To ensure real-time interactions, the waiting time is kept low (3 s), thereby the pre-filter reduces but does not eliminate all sensory errors in the input samples that enter the cognitive model. The cognitive model handles the input samples autonomously. The spoken phrases include content words (nouns, verbs, adverbs) and function words (this, the, a). When the error occurs in the function words, the model can overcome such inaccuracies, because these words do not play a role in constructing the symbolic sentences. The content words identify the semantic of the concept meaning that is learned, either primitive or abstract, and are essential to learning. Therefore, if during speech transcript these words are misrecognised by the robot's speech module, the cognitive system will fail to extract the word that is needed to map to the visual representation during runtime learning or will fail to retrieve appropriate mappings during query. In case of erroneous transcripts of content words, the human repeats the experiment. Conversely, for perfect input samples or irrelevant errors (of function words), the model will generate an output phrase (section Target Training Data). The human supervises the final output content to assess the performance. The accuracy of the model is the correctness in the output, given an error-free sample at the input of the cognitive architecture only (Table 1).

**Table 1:** The number of phrases and the structure of the training/test sets, to validate the generalisation capabilities of the cognitive architecture at run-time.

	**Sample training phrases**	**No. of training phrases**	**Sample test phrases**	**No. of test phrases**
Objects (animals)	_dog dog _frog frog _snake snake	3	_bee bee _elephant elephant	10
Task 1	What is this _dog?	1	What is this _bee?	10
	What kind of animal is this _dog?	1	What kind of animal is this _bee?	10
Task 2	Which one is the dog _dog _frog? Which one is the frog _dog _frog?	4	Which one is the bee _bee _bear?	2·*P*(10, 2) = 180
	Which is a mammal _dog _frog? Which is an amphibian _dog _frog?	4	Which is a mammal _bee _bear? Which is an insect _bee _bear?	2·*P*(10, 2) = 180
Task 3	There are a dog, a frog and a snake You see only _dog _frog Which animal is missing?	6	There are a bee, a bug and a whale You see only _whale _bug Which animal is missing?	*C*(10, 2)·*C*(3, 2) = 360
**TOTAL**		**19**		**750**

*Sample training and test phrases are given*.

The test acquisition tool chain during *manipulation tasks* (Figure 5B) requires high-accuracy of the transcripts to obtain the final higher-order task instruction. As explained above, the human loosely pre-supervises the transcript speech to ensure minimal errors in the input samples. The model can overcome errors of function words, but the instruction is repeated when content words are misrecognised. However, this does not affect the continuation of the tool chain. The failed command is repeated without interrupting the test. This process is smoothed using indicator-words to maintain order sequence (step 1, 2, …). While this introduces a slight limitation, it renders the method more robust to errors. Omitting such indicators does not affect the performance under perfect conditions (perfect samples) but produces cases of erroneous outputs when intermediate failed commands are repeated, hence breaking the test chain. When the misrecognised content words are abstract the model will again fail to generate an output. Though these words do not require memory retrieval, they are stored as a goal. Notice that, during the step-by-step guidance, the human explains what the primitive commands infer by giving auxiliary verbal explanations (e.g., to place the mug, step 3 drop the mug). Therefore, if the goal does not match the verbal explanation (comparison structure, section The Mental Action Sequence), the sequence cannot be generated. This ensures that only the correct abstract words are de-composed to their respective primitive actions and not any random words (notice that the model does not learn words, but how to process words/phrases through the mental actions).

In Figure 5B, the input that goes into the cognitive system is the combination of speech and sensory motor data of the trajectory. Thereby, motor data is fundamental and it solely depends on the robot's autonomous system. When learning new semantics for primitive actions, the robot self-retrieves the corresponding trajectory of the requested action that is determined in its motor map and, extracts its symbolic representation. If the robot does not retrieve the trajectory information correctly, the cognitive model will not be able to process the action information, because it lacks the necessary sensory representation from the motor system. The mapping of the primitive action and its internal motor representation is done without human supervision. These mappings are necessary for the step-by-step composition of higher-order concepts. The backward direction is also true. The cognitive model decomposes higher-order concepts up to the primitive action and the robot maps autonomously the action to its internal motor representation. On the other hand, sensory inaccuracies in the YOLO object tracking do not affect the test process and the performance of the cognitive model (Figure 5B). This is because the performance of the model refers to its capacity to generate the symbolic sequences. These sequences are used to command the robot. The YOLO only tracks the object to determine if and where it is located in the scene. The robot will execute the command if and only it correctly self-maps the action to its internal motor representation of the action AND the object involved matches the detection from YOLO. Otherwise, the requested command will fail. Notice how, motor data extraction for the correct mapping of primitive action semantics to the internal motor map is essential (a) on one end, to generate relevant semantic pairs that are processed by the cognitive model and (b) on the other end, to retrieve the corresponding action from the motor system on human demand (Figure 5B).

All errors occurring during the pre-filtering and those due to imperfect input samples, are a result of the interfaced external components and are not calculated in the total accuracy of the cognitive model. In our specific scenarios, the recognition errors were low given that we used simple commands. Moreover, from an engineering perspective, these errors can be greatly removed with more advanced technical shortcuts. Our main contribution is in implementing the procedural mechanisms to elaborate multimodal information inside the cognitive framework, hence we focused on the errors produced by the model to determine the efficacy of our novel procedural and memory retrieval methodology. In our obtained results, the high test rates indicate that the model can generate the symbolic sequences with high confidence score and decompose higher-order concepts into lower-order concepts successfully, i.e., retrieving the meaning of high-level concepts from primitive concepts and mapping the latter to their internal motor representation. By virtue of the central executive, the system needs not learn the task a priori. Through the mental action sequence, it can autonomously solve similar tasks, in a different workspace and associate sets of primitive actions on relevant objects, to generate the desired outcome.

## Results

**Figure 8:**
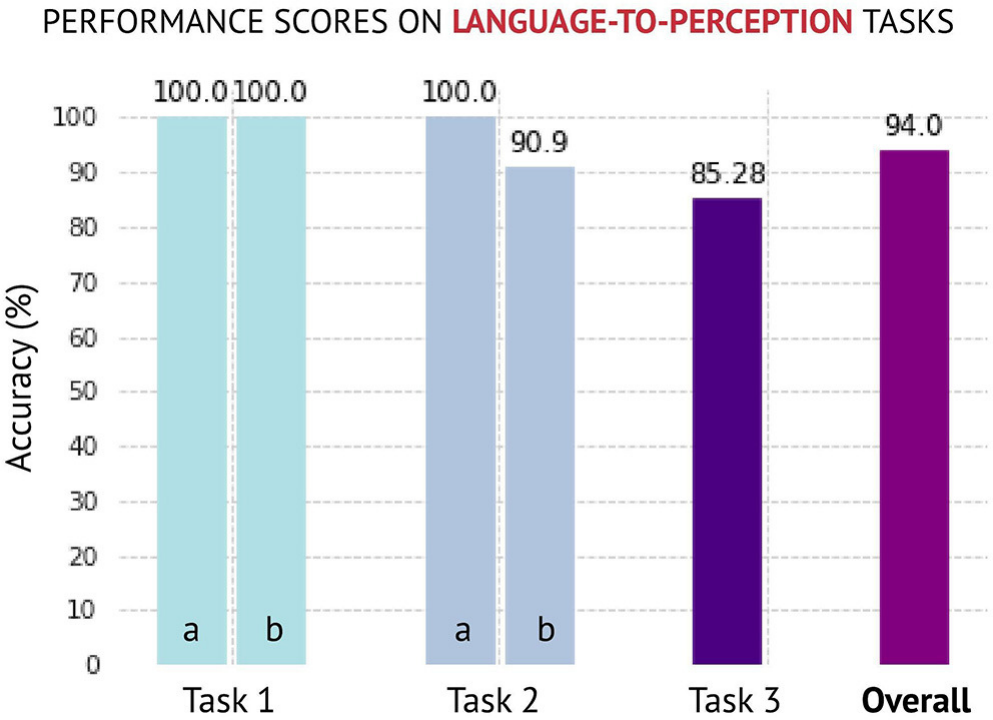
The performance (accuracy) scores obtained for perception tasks. The successful solving of tasks 1–3 highly depends on the model's skill to learn new animals from images at runtime. The phrases acquired during the test stage are similar to those trained but involving the new animals. The system can generalise with an overall accuracy of 94% on novel elements of the animal class, which are not specified in the initial training of the system, without re-training in the whole dataset.

The dataset used in this work contains a limited alphabet of phrases (23 in total, Table 1, Figure 8), necessary to train the basic natural language instructions introduced in perception and manipulation tasks. The test set is acquired gradually during run-time, by adding novel objects and actions to the workspace and new but similar language constructions. To obtain a robust problem-solving system and not an *ad-hoc* neural network that over fits data due to its large-scale, we train our dataset jointly with the original corpus of 1,587 sentences used in Golosio et al. (2015). In the original work, this dataset was used to validate a broad range of language processing functionalities. In this work, we first verified that the original capabilities of the system, to generalise on the tasks trained on the selected thematic groups (Golosio et al., 2015) are preserved. We use the datasets generated here to train the novel tasks jointly in the same state-action association (SAA) of the model and assess a different generalisation property on object and action learning and, language development at run-time. We show and analyse the results of the novel perception and manipulation tasks in the following subsections.

### Perception Tasks

As discussed in section The Robot Learning Experiments, the background knowledge of the model to pre-train tasks 1-3 (target training data) includes only three animals (*dog, frog, snake*). To assess the generalisation skill of the model, we first collect the target test data (section Target Test Data). We aim to verify if the model can scale-up its knowledge continuously after the pre-training, identify cases of data inconsistency in the inputs and its memory and amend its behaviour when the information about the task changes. The desired outcome is that the agent applies novel information acquired during the runtime communication with the human, in the joint workspace, directly and autonomously to solve the required tasks.

To collect the test data, we show to the robot 10 printed images of new animals and dictate the animal names and member classes. The successful execution of this part is essential for solving the tasks 1-3. We construct similar queries to those trained, using the new animals. In Table 1, are given the number and structure of the training and test sets, along with sample phrases. Figure 8 illustrates the accuracy scores obtained for each task. The accuracy metric is the percentage ratio of the correct output phrases over the total requested phrases, where correct phrases are those both semantically and syntactically correct and appropriate for the conversation, e.g., pointing to the correct animal, task 2. The overall accuracy for all language-to-perception tasks (all phrases of the dataset) was 94%. Notice that this accuracy represents the performance of the cognitive model only, where the sample inputs are perfect or insignificantly erroneous (section Handling Sensor-Based Uncertainty).

In the tested examples with the 10 new animals (Task1, Table 1), half were learned directly and half were first queried on, then learned. In the latter case, the model would announce that it did not know the answer. We dictated the correct answer and repeated the query on those animals. In all 5 cases, the model was able to self-modify its answer and reply directly with the correct answer. An example is given below:


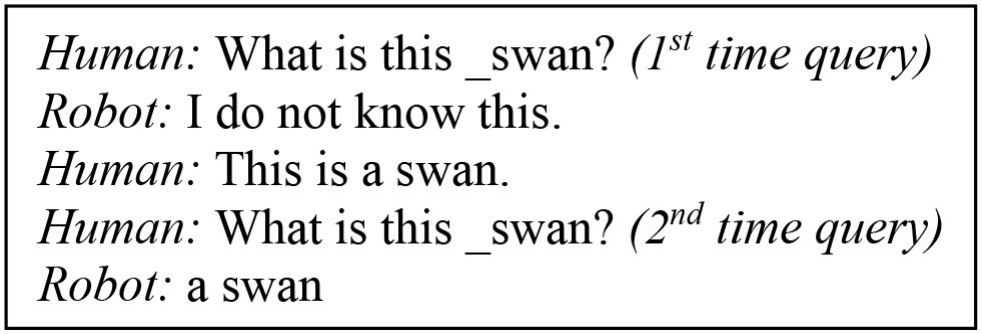


### Manipulation Tasks

We introduced two new tasks similar to “make me a tea,” that are “make me a juice” and “make me a toast.” The former involves the objects *glass, knife* and *orange* and the latter the objects *bread, cheese* and *ham*. We collect the test data as explained in section Target Test Data. First, we show the objects, which the system learns during run-time and, dictate the primitive operations for each object (grasp, release, …), which the robot self-maps to their respective representations in the motor system.

**Figure 9:**
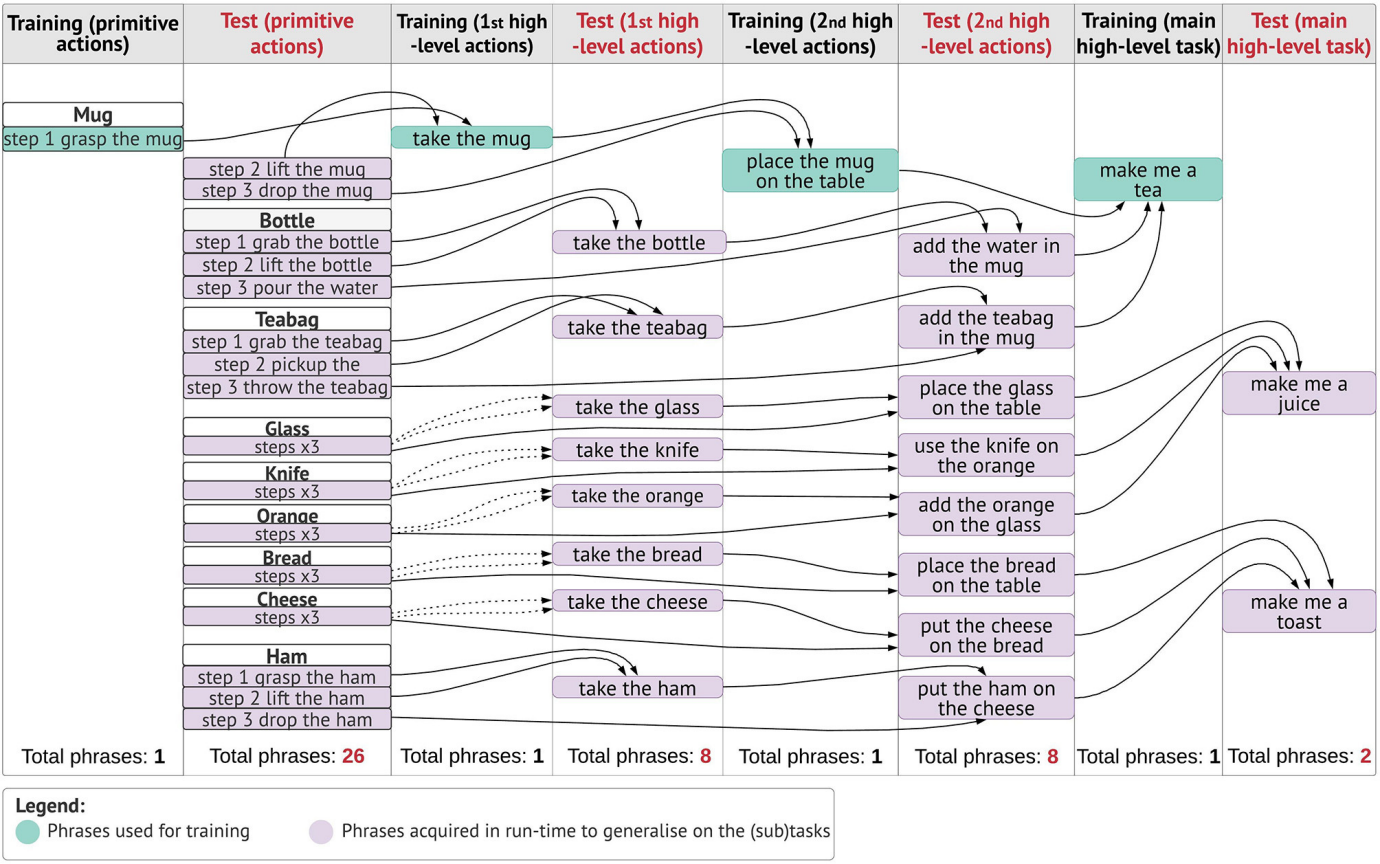
Gradual chain-acquisition of the test set. The model learns new objects and primitive action meanings for those objects. By recalling internal representations of the primitive actions, it converts spoken commands into low-level symbolic sequences. It produces symbolic internal representations for high-level (abstract) by transferring the meanings of primitive actions through the interaction of language with physical exploration. The test set is acquired in sequential steps, by adding similar spoken commands for new objects and involving new actions. As a result, it is verified the ability to learn the semantic meaning of motor primitives and build different internal representations for a given abstract concepts, if the desired outcome and/or the involved tools to reach the outcome have changed (e.g., make tea and make juice).

We guide the model step-by-step to execute elementary actions and construct higher-order commands, e.g., to place the glass on the table in three basic steps (grasp the glass, slide the glass, release the glass), to use the knife on the orange (grab the knife, lift the knife, cut with knife), and so on (Figure 9). The system converts spoken commands into symbolic sequences by recalling its similar past experiences acquired during training, i.e., the mental action sequences. To drive the model's comprehension on the task, the human gives a high-level verbal explanation of what the outcome of the requested primitive behaviours will be. Let us consider how the system learns to use a knife, after having procured the knife (grasp and lift). The human verbally instructs the robot that “to use the knife” the next step is “step 3 cut with knife.” The phrase “to use the knife” is only a spoken utterance used to teach the robot the meaning of the contextual outcome that is expected from the primitive action. The abstract word *use* is not mapped directly to any motor exploration in the robot's internal representation. Instead, the robot's motor map involves only the individual primitive actions of “grasping,” “lifting” and “cutting” an object using pre-determined trajectories (section Target Training Data). When the human commands the robot to “use the knife on the orange,” the central executive of the model generates the mental action sequence that is required to use the knife, by linking iteratively step 3 (cut) to the first two (grasp + lift) and the object involved (orange, for this scenario). The output of the model, i.e., the symbolic representation *_cut* of the action is fed into the robot as a motor command, by autonomous mapping to the respective trajectory in the robot's motor system, which initiates a cutting action on the given object. In this sense, though we start from a static internal representation of the robot, we use its basic motor map to construct and generalise to new high-level actions, not anticipated at design time, and not pre-defined in the robot's motor representation.

For the task “*make me a juice,”* the outcome was:


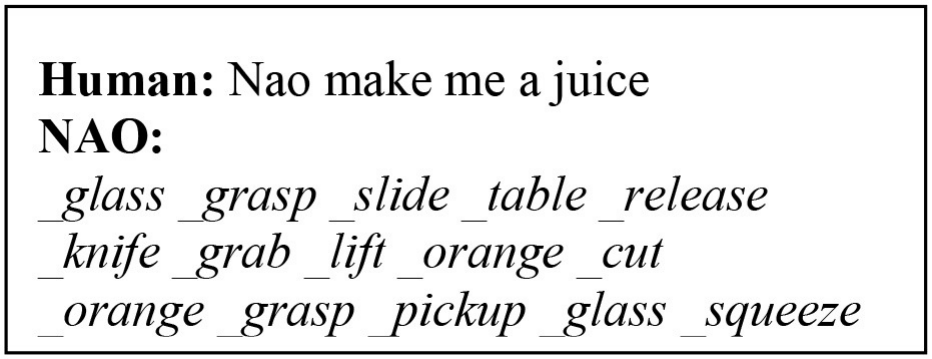


For the task “*make me a toast,”* the outcome was:


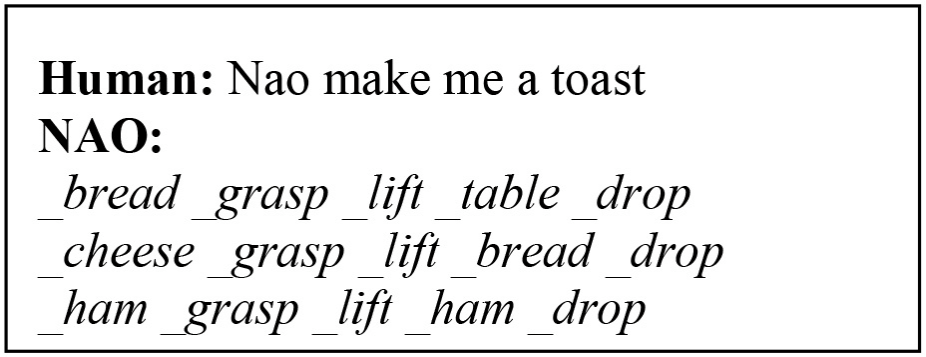


It can be seen from the tested scenarios that the meaning of *make* depends on the outcome of the requested instruction (e.g., *juice* or *toast*), where the outcome involves employing a set of tools (e.g., glass, knife, orange for juice, bread, ham, and cheese for toast) and recalling a series of step-by-step operations acted on these objects (e.g., grasp, lift, release, cut, etc.) (Figure 9).

The test set is constructed incrementally in chain, starting from low-level to higher-order operations (Figure 9). The successful acquisition of primitive actions and their execution dictates if higher-order commands can be performed, given that this is achieved by transferring lower-order meanings, from the interaction of verbal language with physical exploration. Therefore, to execute the main task (*make me a…*), the agent must have successfully solved all intermediate steps in decrementing order (Figure 9), and hooked them appropriately in the motor system. The conducted experiments proved that the agent was able to execute both novel tasks *make me a juice* and *make me a toast*. In Figure 7, the NAO robot performs the task *make me a tea*, using a mug, a bottle and a teabag.

## Discussion

In this work, we proposed a novel methodology to map language to robot perception and behaviour, by implementing appropriate procedural, knowledge representation and memory retrieval mechanisms inside a cognitive framework to elaborate multimodal information from sensorial and linguistic domains. In the learning experiments, we pre-selected (pre-defined) a series of motor primitives and then we allowed the system to self-learn the mapping between the actions and the internal representation corresponding to the actions. Differently, Yamada et al. (2016) investigate and implement a more complex representation learning of these mappings, in a topological structure of word meanings and their compositionality, in a simple task. We preferred using a mediating convention (*_word* string representation) mapping technique instead of multidimensional vectors, to train the long and short-term memory units of our architecture the acquisition of a scale-up vocabulary of concepts, at the cost of a more guided representation of sensory inputs. The whole integrated system was able to interact with the human on several tasks regarding perception and manipulation and, self-handle recognition, information elaboration and production of linguistic and non-linguistic outcomes, in certain contexts. A notable feature of our learning methodology and experiments is that the system learns in runtime from data collected in a rather natural way, with direct teaching of high-level explanations and live captured images of the situated workspace. We demonstrated how the model could continuously scale-up its vocabulary of learned objects and actions meanings and, language constructions, guided by the human, having had only limited innate knowledge on the joint environment.

In our experiments, the sensory data from motor primitives were defined and collected rather artificially, by first defining the actions at the robot end using fixed trajectories. Moreover, all actions are executed for demonstration only and the robot does not have the capability to physically track the object, which it acts upon. The YOLO tracker only determines if the object is present in the workspace, before executing the action. However, we implemented an artifice that allows the robot to execute the same action differently on different objects, according to their features (e.g., grasping a mug vs. grasping a bottle). The final system can be improved significantly, if a tracking module is employed on the robot, to track the object and self-produce the appropriate trajectory of the action on the relevant object. In addition, the link between the action and the robot's internal representation of the action is achieved using a symbolic mapping convention. On one hand, this allows us to represent the sensory input appropriately inside the cognitive model to drive the required procedural mechanisms of multimodal information elaboration and, replace the technical shortcuts flexibly without affecting the working mechanisms inside the cognitive model. For instance, if imitation learning techniques (Hussein et al., 2017) are applied on the robot end, the human user can demonstrate the action, which the robot learns by imitation and maps with the given language utterance. On the pitfall, we do not investigate how the model could naturally learn to predict, amend, and produce trajectories of angle joints according to the scene contextualisation and self-build appropriate internal representations that can be used in our model.

In our error measurement and analysis, we focus mainly on the cognitive model rather than the system as a whole, as we aimed to evaluate the efficacy of the novel procedural and memory retrieval mechanisms that we implement in the framework. However, the performance and stability of the final system is an important consideration. In our specific scenarios, the recognition errors were low given that we used simple phrases and the model's ability to overcome errors occurring in functional words. We applied a pre-selection filter, in which the human loosely supervised the input samples extracted by the recognition modules, to minimise the sensory-based errors. This granted a rather high stability even under noisy laboratory environments (bad lighting, background noise), albeit could introduce a limitation for real-time real-world applications with human users. However, from an engineering perspective, these errors can be greatly reduced with more advanced technical shortcuts or error management methods. An important aspect is that the errors that are not eliminated by the pre-filter do not greatly affect the continuation of tasks, in particular during the tool chain acquisition. The model can continue to build (forward direction) and de-compose (backward direction) higher-order concepts appropriately to handle the requested instruction, without repeating the entire experiment, but only the failed commands in the tool chain.

Finally, through our proposed methodology, we demonstrate a significant learning attribute of the neural cognitive architecture. In a classical neural network, there cannot be added a novel class of data without re-training the whole network. Instead, in our cognitive model, new classes or new elements of classes can be added to the long-term memory without training the entire model anew, using our proposed run-time interactive training methodology, further explained in sections Object Learning and Action Learning on the learning of new objects and actions in the workspace. A classical neural network can learn only if all the elements of the classes are part of the training set. On the contrary, our model is not a classifier, but a complex model that can detect and handle new tasks using mental actions. The model learns the mental action sequence required to solve a certain task, rather than the co-occurrence of words or word combinations. This property is extensively argued in section The Mental Action Sequence.

## Conclusions

In this work, we propose a novel methodology, using a developmental brain-inspired architecture, to model language mapping with visual perceptions and sensorimotor representations in humanoid robots. Through our methodology, we attempt to model the learning of semantic meanings, for perception and manipulation task accomplishment. We encode non-linguistic primitives in symbolic form and process them using similar procedural mechanisms of elaboration of the verbal information. This grants a scalable knowledge representation inside the cognitive architecture, which makes it independent from external coupled devices and effective for incremental open-ended learning. We achieve learning by combining step-by-step natural language descriptions (spoken instructions) and the transferal of lower-order motor meanings (internal state of the agent). We model the acquisition of abstract notions of natural language by indirect mapping in multiple representations, i.e., sensorimotor and linguistic knowledge, for developmental tasks (Cangelosi and Stramandinoli, 2018). Moreover, we propose a method for flexible learning of new objects and actions meanings and language at run-time, which can be directly applied to solve tasks, without re-training the entire model.

During the verification experiments, we obtained an accuracy score of 94% in a relatively large test set (750 phrases) acquired at run-time for *perception tasks*. We demonstrated that the model could successfully represent new primitive action semantics in its motor system and execute high-level commands (abstract action semantics) on demand, by self-generating motor associations, in *manipulation tasks*. Moreover, we showed that the representation of abstract concepts in multiple domains (physical and linguistic) highly depended on the expected outcome of the task and the current setting of the robot's workspace, i.e., the tools to achieve the outcome. The robot was able to perform novel tasks having had no initial knowledge on the objects and actions required to solve the tasks, only by acquiring such knowledge from the interaction with the interlocutor, out of the trained stage.

In future works, we aim to apply more relevant datasets from human-robot interaction corpora to address the compelling problem of language grounding, through an extensive range of experiments. We will explore further signals that affect human-robot communication, such as the role of facial expressions, gestures, intonation, and emotions in achieving a goal task in the human-robot workspace. Moreover, starting from our previous work (Giorgi et al., 2020), where we validate multiple language acquisition using the same framework, we will investigate in-depth the understanding of natural language, by modelling multiple language grounding, with the aim to obtain intelligent agents that comprehend language and tasks, similarly to humans.

## Data Availability Statement

The raw data supporting the conclusions of this article will be made available by the authors, without undue reservation.

## Author Contributions

IG contributed with the design and implementation of the proposed methodology, the drawing of the datasets, the development of the verification experiments, and documentation/analysis of the obtained results. AC and GM contributed with careful supervision of the theoretical relevancy, the experimental work, and with the analysis of the results. All authors contributed to the article and approved the submitted version.

## Conflict of Interest

The authors declare that the research was conducted in the absence of any commercial or financial relationships that could be construed as a potential conflict of interest. The handling Editor declared a past co-authorship with one of the authors AC.

## References

[B1] Alomari M. Duckworth P. Hogg D. Cohn A. (2017). Natural language acquisition and grounding for embodied robotic systems, in Proceedings of the AAAI Conference on Artificial Intelligence, Vol. 31.

[B2] Araki T. Nakamura T. Nagai T. Nagasaka S. Taniguchi T. Iwahashi N. (2012). Online learning of concepts and words using multimodal LDA and hierarchical Pitman-Yor language model, in IEEE/RSJ International Conference on Intelligent Robots and Systems (Vilamoura: IEEE), 1623–1630. 10.1109/IROS.2012.6385812

[B3] Arandjelovic R. Zisserman A. (2017). Look, listen and learn, in Proceedings of the IEEE International Conference on Computer Vision (Venice), 609–617. 10.1109/ICCV.2017.73

[B4] Baddeley A. (2012). Working memory: theories, models, and controversies. Ann. Rev. Psychol. 63, 1–29. 10.1146/annurev-psych-120710-100422 21961947

[B5] Beetz M. Klank U. Kresse I. Maldonado A. Mösenlechner L. Pangercic D.. (2011). Robotic roommates making pancakes, in IEEE-RAS International Conference on Humanoid Robots (Bled), 529–536. 10.1109/Humanoids.2011.6100855

[B6] Borghi A. M. Flumini A. Cimatti F. Marocco D. Scorolli C. (2011). Manipulating objects and telling words: a study on concrete and abstract words acquisition. Front. Psychol. 2:15. 10.3389/fpsyg.2011.00015 21716582 PMC3110830

[B7] Cakmak M. Chao C. Thomaz A. L. (2010). Designing interactions for robot active learners. IEEE Trans. Auton. Ment. Dev. 2, 108–118. 10.1109/TAMD.2010.2051030

[B8] Cangelosi A. (2010). Grounding language in action and perception: from cognitive agents to humanoid robots. Phys. Life Rev. 2, 139–151. 10.1016/j.plrev.2010.02.001 20416855

[B9] Cangelosi A. Schlesinger M. (2018). From babies to robots: the contribution of developmental robotics to developmental psychology. Child Dev. Perspect. 12, 183–188. 10.1111/cdep.12282

[B10] Cangelosi A. Stramandinoli F. (2018). ‘A review of abstract concept learning in embodied agents and robots'. Philos. Trans. R. Soci. B Biol. Sci. 373:20170131. 10.1098/rstb.2017.0131 PMC6015819 29914999

[B11] Cantrell R. Schermerhorn P. Scheutz M. (2011). Learning actions from human-robot dialogues, 20th IEEE International Symposium on Robot and Human Interactive Communication (RO-MAN), 125–130. 10.1109/ROMAN.2011.6005199

[B12] Cowan N. (1998). Attention and Memory: An Integrated Framework. Oxford University Press.

[B13] Cowan N. Rouder J. N. Blume C. L. Saults J. S. (2012). Models of verbal working memory capacity: what does it take to make them work?. Psychol. Rev. 119, 480–499. 10.1037/a0027791 22486726 PMC3618891

[B14] Demiris Y. Khadhouri B. (2006). Hierarchical attentive multiple models for execution and recognition of actions. Robot. Auton. Syst. J. 54, 361–369. 10.1016/j.robot.2006.02.003

[B15] Dubba K. S. De Oliveira M. R. Lim G. H. Kasaei H. Lopes L. S. Tome A.. (2014). Grounding language in perception for scene conceptualization in autonomous robots, in Qualitative Representations for Robots: Papers from the AAAI Spring Symposium (Palo Alto, CA), 26–33.

[B16] Duffy B. R. Joue G. (2000). Intelligent robots: the question of embodiment, in Proceedings of the Brain-Machine Workshop (Nashville, TN).

[B17] Elman J. (1991). Distributed representations, simple recurrent networks, and grammatical structure. Mach. Learn. 7, 195–225. 10.1007/BF00114844

[B18] Feldman J. Narayanan S. (2004). Embodied meaning in a neural theory of language. Brain Lang. 89, 385–392. 10.1016/S0093-934X(03)00355-9 15068922

[B19] Giorgi I. Golosio B. Esposito M. Cangelosi A. Masala G. L. (2020). Modelling multiple language learning in a developmental cognitive architecture, in IEEE Transactions on Cognitive and Developmental Learning. 10.1109/TCDS.2020.3033963

[B20] Glenberg A. Kaschak K. (2002). Grounding language in action. Psychon. Bull. Rev. 9, 558–565. 10.3758/BF03196313 12412897

[B21] Golosio B. Cangelosi A. Gamotina O. Masala G. (2015). A cognitive neural architecture able to learn and communicate through natural language. PLoS ONE 10:e0140866. 10.1371/journal.pone.0140866 26560154 PMC4641699

[B22] Gu S. Holly E. Lillicrap T. Levine S. (2017). Deep reinforcement learning for robotic manipulation with asynchronous off-policy updates, in IEEE International Conference on Robotics and Automation (ICRA) (Singapore), 3389–3396. 10.1109/ICRA.2017.7989385

[B23] Heinrich S. Wermter S. (2018). Interactive natural language acquisition in a multi-modal recurrent neural architecture. Connect. Sci. 30, 99–133. 10.1080/09540091.2017.1318357

[B24] Heinrich S. Yao Y. Hinz T. Liu Z. Hummel T. Kerzel M.. (2020). Crossmodal language grounding in an embodied neurocognitive model. Front. Neurorobot. 14:52. 10.3389/fnbot.2020.00052 33154720 PMC7591775

[B25] Hinaut X. Petit M. Pointeau G. Dominey P. F. (2014). Exploring the acquisition and production of grammatical constructions through human-robot interaction with echo state networks. Front. Neurorobot. 8:16. 10.3389/fnbot.2014.00016 24834050 PMC4018555

[B26] Hinaut X. Twiefel J. (2019). Teach Your Robot Your Language! trainable neural parser for modeling human sentence processing: examples for 15 languages, in IEEE Transactions on Cognitive and Developmental Systems. Vol. 12, 179–188. 10.1109/TCDS.2019.2957006

[B27] Hinaut X. Wermter S. (2014). An incremental approach to language acquisition: thematic role assignment with echo state networks, in Artificial Neural Networks and Machine Learning – ICANN 2014. Lecture Notes in Computer Science. Vol. 8681 eds Wermter S. S.. (Cham: Springer). 10.1007/978-3-319-11179-7_5

[B28] Hussein A. Gaber M. M. Elyan E. Jayne C. (2017). Imitation learning: a survey of learning methods. ACM Comput. Surv. 50:35. 10.1145/3054912

[B29] Iwahashi N. (2008). Interactive learning of spoken words and their meanings through an audio-visual interface. IEICE Trans. Inf. Syst. 91, 312–321. 10.1093/ietisy/e91-d.2.312

[B30] Jamone L. Ugur E. Cangelosi A. Fadiga L. Bernardino A. Piater J.. (2018). Affordances in psychology, neuroscience, and robotics: a survey. IEEE Trans. Cogn. Dev. Syst. 10, 4–25. 10.1109/TCDS.2016.2594134

[B31] Krizhevsky A. Sutskever I. Hinton G. E. (2012). ImageNet classification with deep convolutional neural networks. Adv. Neural Inform. Proces. Syst. 25, 1097–1105. 10.1145/3065386

[B32] Kurup U. Lebiere C. (2012). What can cognitive architectures do for robotics. BICA 2, 88–99. 10.1016/j.bica.2012.07.004

[B33] Levine S. Finn C. Darrell T. Abbeel P. (2016). End-to-end training of deep visuomotor policies. J. Mach. Learn. Res. 17, 1334–1373. Available online at: http://jmlr.org/papers/v17/15-522.html

[B34] Matuszek C. FitzGerald N. Zettlemoyer L. Bo L. Fox D. (2012). A joint model of language and perception for grounded attribute learning, in Proceedings of the 29th International Conference on Machine Learning (ICML'12) (Madison, WI), 1435–1442.

[B35] McClelland J. Kawamoto A. (1986). Mechanisms of sentence processing: Assigning roles to constituents of sentences, in Parallel Distributed Processing. Explorations in the Microstructure of Cognition, vol. 2, eds McClelland J. L. Rumelhart D. E. (Cambridge, MA: MIT Press), 272–325.

[B36] Mealier A. L. Pointeau G. Mirliaz S. Ogawa K. Finlayson M. Dominey P. F. (2017). Narrative constructions for the organization of self experience: proof of concept via embodied robotics. Front. Psychol. 8:1331. 10.3389/fpsyg.2017.01331 28861011 PMC5559541

[B37] Miikkulainen R. (1993). Subsymbolic Natural Language Processing: An Integrated Model of Scripts, Lexicon, and Memory. Cambridge, MA: MIT Press.

[B38] Miyazawa K. Horii T. Aoki T. Nagai T. (2019). Integrated cognitive architecture for robot learning of action and language. Front. Robot. AI 6:131. 10.3389/frobt.2019.00131 33501146 PMC7805838

[B39] Morse F. A. Cangelosi A. (2017). Why are there developmental stages in language learning? a developmental robotics model of language developments. Cogn. Sci. 41 (Suppl. 1), 32–51. 10.1111/cogs.12390 27680660

[B40] Moulin-Frier C. Fischer T. Petit M. Pointeau G. Puigbo J. Y. Pattacini U.. (2017). DAC-h3: a proactive robot cognitive architecture to acquire and express knowledge about the world and the self. IEEE Trans. Cogn. Dev. Syst. 10, 1005–1022. 10.1109/TCDS.2017.2754143

[B41] Nyga D. Roy S. Paul R. Park D. Pomarlan M. Beetz M.. (2018). Grounding robot plans from natural language instructions with incomplete world knowledge, in Proceedings of the 2nd Conference on Robot Learning (CoRL) (Zürich), 714–723.

[B42] Ogata T. Murase M. Tani J. Komatani K. Okuno H. G. (2007). Two-way translation of compound sentences and arm motions by recurrent neural networks, in IEEE/RSJ International Conference on Intelligent Robots and Systems (San Diego, CA: IEEE), 1858–1863. 10.1109/IROS.2007.4399265

[B43] Ogata T. Sugano S. Tani J. (2005). Open-end human-robot interaction from the dynamical systems perspective: mutual adaptation and incremental learning. Adv. Robot. 19, 651–670. 10.1163/1568553054255655

[B44] Palm G. (1990). Cell assemblies as a guideline for brain research. Concepts Neurosci. 1, 133–147.

[B45] Pecher D. Zwaan R. A. (eds.) (2005). Grounding Cognition: The Role of Perception and Action in Memory, Language, and Thinking. Cambridge: Cambridge University Press. 10.1017/CBO9780511499968

[B46] Perlovsky L. I. (2009). Language and cognition. Neural Networks 22, 247–257. 10.1016/j.neunet.2009.03.007 19419838

[B47] Pulvermüller F. Garagnani M. (2014). From sensorimotor learning to memory cells in prefrontal and temporal association cortex: a neurocomputational study of disembodiment. Cortex 57, 1–21. 10.1016/j.cortex.2014.02.015 24769063

[B48] Recupero D. R. Spiga F. (2019). Knowledge acquisition from parsing natural language expressions for humanoid robot action commands. Inform. Proces. Manage. 57:102094. 10.1016/j.ipm.2019.102094

[B49] Redmon J. Divvala S. Girshick R. Farhadi A. (2016). You only look once: unified, real-time object detection, 2016 IEEE Conference on Computer Vision and Pattern Recognition (CVPR) (Las Vegas, NV), 779–788. 10.1109/CVPR.2016.91

[B50] Roy D. (2003). Learning visually-grounded words and syntax for a scene description task. Comput. Speech Lang. 16, 353–385. 10.1016/S0885-2308(02)00024-4

[B51] Roy D. Hsiao K. Y. Mavridis N. Gorniak P. (2003). Ripley, hand me the cup! (sensorimotor representations for grounding word meaning), in 2003 IEEE Workshop on Automatic Speech Recognition and Understanding (IEEE Cat. No.03EX721) (St Thomas, VI).

[B52] Roy D. Pentland A. (2002). Learning words from sights and sounds: a computational model. Cogn. Sci. 26, 113–146. 10.1207/s15516709cog2601_4

[B53] Sabinasz D. Richter M. Lins J. Schöner G. (2020). Grounding spatial language in perception by combining concepts in a neural dynamic architecture in Proceedings of 42nd Annual Conference of the Cognitive Science Society (Toronto, ON).

[B54] Sharkey N. Zeimke T. (2000). Life, mind and robots: the ins and outs of embodied cognition, in Symbolic and Neural Net Hybrids, eds Wermter S. Sun R. (Berlin; Heidelberg: MIT Press). 10.1007/10719871_22

[B55] She L. Cheng Y. Chai J Y. Jia Y. Yang S. Xi N. (2014). Teaching robots new actions through natural language instructions, The 23rd IEEE International Symposium on Robot and Human Interactive Communication (Edinburgh), 868–873. 10.1109/ROMAN.2014.6926362

[B56] SoftBank Robotics (2021). NAO the Humanoid Programmable Robot. Available online at: https://www.softbankrobotics.com/emea/en/nao (accessed April 21, 2021).

[B57] Steels L. (2003). Evolving grounded communication for robots. Trends Cogn. Sci. 7, 308–312. 10.1016/S1364-6613(03)00129-3 12860189

[B58] Štepánová K. Klein F. B Cangelosi A Vavreèka M, (2018). Mapping language to vision in a real-world robotic scenario. IEEE Trans. Cogn. Dev. Syst. 10, 784–794. 10.1109/TCDS.2018.2819359

[B59] Stoytchev A. (2008). Learning the affordances of tools using a behavior-grounded approach, in Towards Affordance-Based Robot Control, eds Rome E. Hertzberg J. Dorffner G. (Berlin; Heidelberg. Springer), 140–158. 10.1007/978-3-540-77915-5_10

[B60] Stramandinoli F. Marocco D. Cangelosi A. (2012). The grounding of higher order concepts in action and language: a cognitive robotics model. Neural Networks 32, 165–173. 10.1016/j.neunet.2012.02.012 22386502

[B61] Sugita Y. Tani J. (2005). Learning semantic combinatoriality from the interaction between linguistic and behavioral processes. Adapt. Behav. 13, 33–52. 10.1177/105971230501300102

[B62] Tani J. (2016). Exploring Robotic Minds: Actions, Symbols, and Consciousness As Self-Organizing Dynamic Phenomena, 1st Edn. New York, NY: Oxford University Press, Inc. 10.1093/acprof:oso/9780190281069.001.0001

[B63] Tellex S. Thaker P. Joseph J. Roy N. (2014). Learning perceptually grounded word meanings from unaligned parallel data. Mach. Learn. 94, 151–167 10.1007/s10994-013-5383-2

[B64] Thomaz A. L. Cakmak M. (2009). Learning about objects with human teachers, in Proceedings of the 4th ACM/IEEE International Conference on Human Robot Interaction, HRI '09 (La Jolla, CA), 15–22. 10.1145/1514095.1514101

[B65] Twiefel J. Hinaut X. Borghetti M. Strahl E. Wermter S. (2016). Using natural language feedback in a neuro-inspired integrated multimodal robotic architecture, in 2016 25th IEEE International Symposium on Robot and Human Interactive Communication (RO-MAN) (New York, NY: IEEE), 52–57. 10.1109/ROMAN.2016.7745090

[B66] Wilson M. (2002). Six views of embodied cognition. Psych. Bull. Rev. 9, 625–636. 10.3758/BF03196322 12613670

[B67] Yamada T. Murata S. Arie H. Ogata T. (2015). Attractor representations of language-behavior structure in a recurrent neural network for human-robot interaction, in IEEE/RSJ International Conference on Intelligent Robots and Systems (IROS2015) (Hamburg: IEEE), 4179–4184. 10.1109/IROS.2015.7353968

[B68] Yamada T. Murata S. Arie H. Ogata T. (2016). Dynamical integration of language and behavior in a recurrent neural network for human–robot interaction. Front. Neurorobot. 10:5. 10.3389/fnbot.2016.00005 27471463 PMC4946379

[B69] Yamada T. Murata S. Arie H. Ogata T. (2017). Representation learning of logic words by an RNN: from word sequences to robot actions. Front. Neurorobot. 11:70. 10.3389/fnbot.2017.00070 29311891 PMC5744442

[B70] Yang S. Gao Q. Liu C. Xiong C. Zhu S. C. Chai J Y. (2016). Grounded semantic role labeling, in Proceedings of the 15th Annual Conference of the North American Chapter of the Association for Computational Linguistics: Human Language Technologies (NAACL-HLT) (San Diego, CA), 149–159. 10.18653/v1/N16-1019

[B71] Yu H. Siskind J. M. (2013). Grounded language learning from video described with sentences, in Proceedings of the 51st Annual Meeting of the Association for Computational Linguistics (ACL),53–63.

